# Substance use, criminal behaviour and psychiatric symptoms following childhood traumatic brain injury: findings from the ALSPAC cohort

**DOI:** 10.1007/s00787-017-0975-1

**Published:** 2017-03-17

**Authors:** Eleanor Kennedy, Jon Heron, Marcus Munafò

**Affiliations:** 1 MRC Integrative Epidemiology Unit at the University of Bristol and School of Experimental Psychology, University of Bristol, Bristol, United Kingdom; 2 School of Social and Community Medicine, University of Bristol, Bristol, United Kingdom; 3 School of Experimental Psychology, 12a Priory Road, Bristol, BS8 1TU United Kingdom

**Keywords:** Traumatic brain injury, Head injury, Risk behaviour, Crime, Substance use

## Abstract

Recent research suggests a link between traumatic brain injury (TBI) in youth and later risk behaviour. We explored the association between mild TBI and psychiatric symptoms, substance use and criminal behaviour using data from a longitudinal birth cohort. Participants with mild TBI (*n* = 800), orthopaedic injuries (*n* = 2305) and no injuries (*n* = 8307) were identified from self and parent reports up to age 16 years. Self-report measures of substance use (alcohol, tobacco and cannabis) and criminal behaviours, and parent-reported psychiatric symptoms were collected at age 17 years. Analyses were adjusted for pre-birth and early childhood confounders. Participants with a TBI showed increased odds of hazardous alcohol use compared to those with no injury and those with an orthopaedic injury. Relative to those with no injury, participants with a TBI showed increased odds of problematic use of tobacco and cannabis, being in trouble with the police and having more parent-reported conduct problems. Sustaining either a TBI or an orthopaedic injury increased the odds of offending behaviour compared to having no injuries. There was no clear evidence of association between orthopaedic injury and the other risk outcomes. The increased odds of risk behaviour associated with TBI relative to no injury replicated previous research. However, the inclusion of a non-brain-related injury group adds evidence for a possible causal pathway between mild TBI in youth and later hazardous alcohol use only. This highlights the importance of including an additional negative control injury group in mild TBI research.

**Electronic supplementary material:**

The online version of this article (doi:10.1007/s00787-017-0975-1) contains supplementary material, which is available to authorized users.

## Introduction

Traumatic brain injury (TBI) is the leading cause of disability in young people [1]. A recent review of paediatric TBI estimated that each year TBI affects over 3 million children worldwide, and that over 80% of these are mild [2]. The definition of what constitutes a mild TBI varies between studies; however, the Centre for Disease Control suggests the following: “Mild TBI is an injury to the head (arising from blunt trauma or acceleration or deceleration forces) that results in one or more of the following: any period of confusion, disorientation, or impaired consciousness; any dysfunction of memory around the time of injury; loss of consciousness lasting less than 30 min; or the onset of observed signs or symptoms of neurological or neuropsychological dysfunction” [3]. Previous cross-sectional research has shown increased substance use [4], disruptive behaviour disorders [5], school violence [6] and conduct problems [7, 8] in participants with a history of mild TBI.

Evidence for a prospective association between mild TBI and negative behavioural outcomes comes from three cohort studies. In a sample of over one million Swedish people, having a TBI registered with hospital before age 25 years was associated with increased risk of adverse outcomes, including drawing a disability pension, psychiatric visit or hospitalisation, low educational attainment, and welfare recipiency. For those with a mild TBI, the risk ratios for these outcomes ranged from 1.18 to 1.52 [9]. Findings from the Northern Finland 1966 Birth Cohort Study indicate that a mild TBI before age 14 years was associated with drinking to intoxication at age 14 years [10]. In the same cohort, male participants with a TBI before age 15 years were at higher risk of committing a crime registered with the Ministry of Justice from ages 16 to 31 years, and those with a TBI had a twofold increased risk of developing a psychiatric disorder, which increased to fourfold for criminality combined with a psychiatric disorder [11]. In the Christchurch Health and Development Study (CHDS), participants who had experienced a mild TBI requiring an inpatient hospital stay between birth and age five years had higher self- and parent-ratings of conduct disorder/oppositional defiant disorder and substance abuse at age 14–16 years [12], and a higher likelihood of alcohol and drug dependence at age 16–25 years, which mediated a relationship between the same injury and an increased number of arrests, property offences and violent offences [13]. Any mild TBI at age 6–15 years was linked with increased arrests and property offences at age 16–25 years, hospitalisation for the injury was additionally associated with violent offences [13].

In this study, we investigated the association between mild TBI and risk behaviour in a United Kingdom birth cohort. TBI was based on incidences of skull fracture and loss of consciousness due to a head injury reported by parents and children at multiple time points up to age 16 years. Risk behaviour was defined as psychiatric symptoms, substance use, and criminal behaviours. To strengthen causal inference we incorporated a negative control exposure group, where confounding structures are likely to be similar but there is no pathway between the exposure and the outcome [14]. If the observed association is larger for exposure of interest than for the negative control exposure this increases confidence that the association may be causal [15]. Previously, in the Swedish population study, individuals who sustained non-TBI fall-related injuries were less likely to have poor adult outcomes than those with a TBI before age 25 years [9]. Additionally, Fazel and colleagues found that participants with a history of epilepsy were less likely to commit violent crime than those who sustained a TBI [16]. In our study, participants with a history of fracture or broken bone formed the negative control exposure group as this type of injury has a similar confounding structure to TBI but lacks the plausible biological mechanism (i.e., brain injury) for an association with risk behaviour. The effect of age at injury was investigated in secondary analyses separating the cohort into those with childhood injuries and those with adolescent injuries.

## Methods

### Participants

Participants were drawn from a longitudinal birth cohort study, the Avon Longitudinal Study of Parents and Children (ALSPAC). Initially 14,541 pregnant women who were expected to give birth between 1 April 1991 and 31 December 1992 were recruited into the study in the South West region of England [17]. The study website contains details of all data available through a fully searchable data dictionary (http://www.bris.ac.uk/alspac/researchers/data-access/data-dictionary/). Ethics approval for the study was obtained from the ALSPAC Ethics and Law Committee and the Local Research Ethics Committees.

### Measures

*Injury groups* In the ALSPAC questionnaires, parents were asked if their child had incurred any injuries across a range of ages up to 11 years. Similar self-report questionnaires were completed by the offspring; at age 15 years participants reported on fractures incurred since their 12th birthday, including skull fractures, and at age 16 years participants reported on a head injury since their 14th birthday or fractures in the last 6 months. Information was gathered from a series of postal questionnaires. A positive response to the item “head injury resulting in a loss of consciousness” or the item “cracked or broke skull” was used to identify participants in the mild TBI group. A positive response to any of the items “broke arm or hand”, “broke leg or foot” or “broken other bone” was used to identify participants in the orthopaedic injury (OI) control group. Participants who incurred both a head injury and a broken bone were included in the TBI group only. Participants for whom there were no positive responses to the above items were included in the no injury control group. For the secondary analyses, participants were assigned to either a childhood or an adolescent injury group based on the age at which their first injury occurred.

*Substance use* Data on tobacco, alcohol and cannabis use was gathered by a self-report questionnaire at age 17 years. Problematic use was assessed at age 17 years using the Fagerström Test for Nicotine Dependence (FTND) [18], Alcohol Use Disorders Identification Test (AUDIT) [19], and the Cannabis Abuse Screening Test (CAST) [20]. Responses were used to create category variables for each substance. The FTND is a six-item scale with total scores ranging from 0 to 10; the tobacco variable contained the levels: “not regular smoker”, “weekly smoker” and “FTND score of over 4”. The AUDIT consists of ten items with total score ranging from 0 to 40, we used a cut-off score of 8 to identify hazardous drinkers. The alcohol use variable contained the levels “non-hazardous use” and “hazardous use”. The CAST is a four item scale with a total score range from 0 to 6; cannabis use was categorised as “not used in the last 12 months”, “used in the last 12 months” and “CAST score of one or more”. Conservative cut-off scores were used to define problematic use to reflect the young age of the participants.

*Criminal behaviour* A self-report questionnaire at age 17 years was used to assess criminal behaviour in terms of offences committed and trouble with the police [21]. Participants were classified as either having committed “no offences”, “at least one non-violent offence” or “at least one violent offence” based on questions relating to behaviours such as theft, assault and property damage. There was a single item asking if the participant had “sold illegal drugs to someone” within this questionnaire. A second variable related to whether or not a participant had ever been in trouble with the police was included with the levels “never”, “in trouble with the police with no conviction” and “one or more criminal record offence”.

*Psychiatric symptoms* Parents completed one measure of psychiatric symptoms, the Strengths and Difficulties Questionnaire (SDQ), while the offspring completed the Development and Well-Being Assessment (DAWBA). The SDQ [22] is a 25-item parent-rated scale; each item can be rated as ‘not true’, ‘somewhat true’ or ‘certainly true’. There are ten strengths, fourteen difficulties and one neutral item within five subscales. Parents completed the entire SDQ; however, only two of the subscales assessing conduct problems and peer problems at age 16 years were included in the current analysis. The DAWBA [23] is a semi-structured interview administered to the offspring at age 15 years. The interview contains sections measuring symptoms of various emotional, behavioural and hyperactivity disorders with skip-rules. The questions are designed to closely follow the diagnostic criteria for psychiatric disorders as defined by the Diagnostic and Statistical Manual of Mental Disorders, 4th Edition (DSM-IV) or the International Classification of Diseases (ICD-10). A composite variable of externalising disorder symptoms, as well as individual variables relating to diagnoses of oppositional defiant disorder (ODD), conduct disorder (CD) and attention deficit hyperactivity disorder (ADHD) at age 15 years were included as variables in the secondary analysis on childhood injuries.

*Confounders* Models were adjusted for confounders that preceded the TBI measurements and were previously shown to have associations with TBI. Confounders considered included: (1) pre-birth confounders (mother’s age and education at birth [24], social class (based on either the paternal or maternal self-reported highest occupation level related to the Registrar General’s classification of occupations) and gender), and (2) childhood confounders (early life events [24], parenting style (based on self-report questionnaires assessing parental bonding, positive and negative parenting experiences) [24], maternal alcohol use [10] and maternal tobacco smoking). Tobacco, alcohol and cannabis were mutually adjusted for by including these variables as covariates in the final adjustment model.

### Statistical analysis

Ordinal regression was used to explore the association between childhood injuries from birth to age 16 years, and the three-level variables relating to substance use (tobacco and cannabis) and criminal behaviour (offences, trouble with the police) at age 17 years. The gologit2 command [25] was used to permit testing for the more parsimonious proportional odds model (PO). We first, for the univariable model consisting of outcome and exposure, compared constrained (PO) and unconstrained (non-PO) models using a likelihood ratio test, accepting the simpler model if the *p* value was greater than 0.01. Next, confounders were included without the PO restriction for these additional covariates. Finally, support for PO for the exposure was re-examined within these multivariable models.

Logistic regression was used to explore the association between childhood injuries and the two-level variables relating to substance use (alcohol) and psychiatric symptoms. Separate secondary analyses were conducted using childhood injuries, sustained between birth and age 11 years, and adolescent injuries, sustained between age 12 and age 16, to explore the impact of age at injury.

The impact of confounders on the relationship between TBI and risk behaviours was explored by comparing unadjusted estimates with those adjusted for pre-birth variables (model 1) and those further adjusted for childhood variables (model 2). Substance use and crime frequently co-occur. To explore the impact this relationship may have on the main association of interest, an additional model adjusted for other substance use variables (model 3) was conducted for analyses of each of the substance use and crime variables. This model included adjustment for crime variables in the analyses on substance use. As each level of adjustment increases, the sample size decreases as those with missing data are excluded from the analysis. As a sensitivity analysis, all analyses were conducted on the full sample and then conducted on only those participants with complete data (i.e., complete cases). Comparisons were made between the no injury controls and each injury group, and also directly between the TBI group and the OI group. For the comparison between the TBI and OI groups, additional sensitivity analyses were conducted excluding participants who had incurred both a TBI and OI. Analyses were conducted using Stata version 13 (StataCorp LP, Texas).

## Results

### Characteristics of participants

**Fig. 1:**
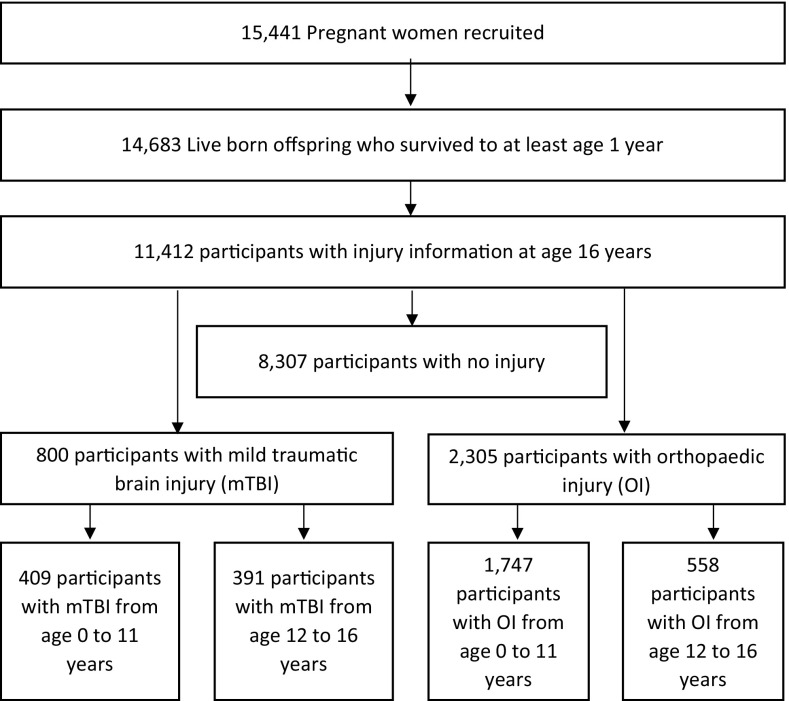
Flow chart of final sample

Descriptive statistics for the sample are shown in Table 1 and a flow chart of the final sample in Fig. 1. Between birth and age 16 years, there were 800 participants with a reported TBI (57% male), 2305 participants with a reported OI (56% male) and 8307 participants with neither injury reported (50% male). There were 289 participants included in the TBI groups who had incurred both a TBI and an OI. There were 56 participants who experienced more than one TBI. Participants with a TBI were more likely to be male and to have more adverse early life events. Unexpectedly, individuals with no reported injury were more likely to come from a low income family and to live in rented subsidised housing; their mothers had a lower level of education and were on average six months younger than mothers of children with TBI.

**Table 1:** Descriptive statistics for covariates; injuries from birth to age 16 years

	No injury	TBI	OI	*p* value*
	(*n* = 8307)	(*n* = 800)	(*n* = 2305)	
	*N* (%)	*N* (%)	*N* (%)	
Male	4109 (49.5)	457 (57.1)	1283 (55.7)	<0.001
Social class IV–V^a^	3052 (42.9)	273 (37.7)	786 (39.5)	0.001
Rented subsidised housing	967 (12.5)	60 (8.0)	181 (8.5)	<0.001
Mother completed secondary school	4826 (63.4)	434 (57.7)	1236 (58.9)	<0.001
Maternal daily smoking	2246 (28.6)	212 (27.6)	576 (26.7)	0.186
Maternal daily alcohol use	989 (12.6)	110 (14.3)	309 (14.3)	0.067
Three or more early life events^b^	4107 (52.9)	470 (61.6)	1220 (57.1)	<0.001

	M (SD)	M (SD)	M (SD)	
Maternal age at birth (years)	28.42 (4.76)	28.92 (4.76)	28.72 (4.74)	0.001
Bonding at 8 months^c^	28.25 (3.68)	28.08 (3.55)	28.20 (3.59)	0.512
Positive parenting experience at 21 months^d^	5.99 (1.51)	6.01 (1.53)	6.00 (1.55)	0.934
Negative parenting experience at 21 months^d^	20.80 (2.74)	20.63 (2.84)	20.77 (2.73)	0.281

*TBI* traumatic brain injury, *OI* orthopaedic injury

* *p* values calculated using Chi square or analysis of variance

^a^Highest social class of either parent is skilled non-manual or lower occupation based on the Registrar General’s classification of occupations

^b^Parent-reported questionnaire relating to upsetting events in the child’s life completed when offspring was 6, 30, 42 and 81 months old

^c^Parent-report questionnaire completed when offspring was 8 months old

^d^Positive and negative parenting experiences based on parent-completed questionnaire when offspring was 21 months old

### Associations with alcohol, tobacco and cannabis use

Individuals with TBI were at increased odds of hazardous use of alcohol (unadjusted OR = 1.51, 95% CI 1.21–1.90), problematic use of tobacco (unadjusted OR = 1.47, 95% CI 1.12–1.94) and problematic use of cannabis (unadjusted OR = 1.54, 95% CI 1.22–1.94). These associations were robust to adjustment for pre-birth and childhood confounders. Mutual adjustment for the other substance use variables weakened the associations of TBI with alcohol and cannabis use, and fully attenuated the association with tobacco use. In the negative control analyses, OI was associated with cannabis use (unadjusted OR = 1.22, 95% CI 1.04–1.43), but this was attenuated following adjustment for pre-birth and childhood confounders. There was no evidence for any associations between OI and alcohol or tobacco use, implying that the associations observed are specific to TBI. In the direct comparison to those with OI, participants with TBI were at increased odds of hazardous alcohol use only (unadjusted OR 1.34, 95% CI 1.05–1.72). These results are shown in Table 2. Excluding participants with both TBI and OI strengthened the association between TBI and alcohol use (unadjusted OR 1.48, 95% CI 1.10–1.99; adjusted OR 1.57, 95% CI 1.13–2.18); findings from this analysis can be seen in Supplementary Table S4 and S5. The findings from the complete case analyses did not differ substantially and can be seen in Supplementary Table S3.

**Table 2:** Associations between traumatic brain injury and orthopaedic injuries from birth to age 16 years and substance use at age 17 years

Substance use	OR (95% CI)			
	Unadjusted	Model 1	Model 2	Model 3
Alcohol^a,c^				
*n*	3694	3263	2884	2074
TBI vs no injury	1.51 (1.21–1.90)	1.46 (1.15–1.85)	1.56 (1.21–2.01)	1.31 (0.94–1.82)
OI vs no injury	1.13 (0.97–1.31)	1.06 (0.90–1.25)	1.06 (0.89–1.27)	0.77 (0.61–0.98)
TBI vs OI	1.34 (1.05–1.72)	1.37 (1.06–1.79)	1.47 (1.11–1.94)	1.69 (1.17–2.45)
Omnibus *p*	0.045	0.265	0.251	0.080
Tobacco^b,d^				
*n*	3099	2741	2420	2074
TBI vs no injury	1.47 (1.12–1.94)	1.51 (1.12–2.03)	1.46 (1.06–2.01)	1.09 (0.74–1.62)
OI vs no injury	1.16 (0.96–1.42)	1.20 (0.97–1.49)	1.22 (0.97–1.54)	1.15 (0.86–1.55)
TBI vs OI	1.26 (0.93–1.72)	1.26 (0.91–1.74)	1.19 (0.84–1.70)	0.95 (0.61–1.47)
Omnibus *p*	0.060	0.044	0.050	0.331
Cannabis^b,e^				
*n*	3979	3505	3090	2074
TBI vs no injury	1.54 (1.22–1.94)	1.36 (1.06–1.75)	1.39 (1.07–1.80)	1.23 (0.87–1.74)
OI vs no injury	1.22 (1.04–1.43)	1.15 (0.97–1.37)	1.15 (0.96–1.39)	1.02 (0.79–1.33)
TBI vs OI	1.26 (0.98–1.62)	1.18 (0.90–1.55)	1.20 (0.90–1.60)	1.20 (0.82–1.77)
Omnibus *p*	0.004	0.054	0.071	0.718

Sample size reduces per adjustment as the participants who are missing covariate data get excluded

*TBI* traumatic brain injury, *OI* orthopaedic injury, *Unadjusted Injuries* from birth to age 16 years with main substance use variable in each analysis, *Model 1* As unadjusted with additional adjustment for pre-birth confounders (mother’s age at birth, mother’s education at birth, social class and gender), *Model 2* As Model 1 with additional adjustment for childhood confounders (early life events, parental bonding, positive and negative parenting experiences, maternal alcohol use and maternal tobacco smoking), *Model 3* As Model 2 with additional adjustment for substance use and crime variables

^a^Logistic regression

^b^Generalised ordinal regression

^c^Alcohol measured using the Alcohol Use Disorder Identification Test (AUDIT)

^d^Tobacco measured using the Fagerström Test for Nicotine Dependence

^e^Cannabis measured using the Cannabis Abuse Screening Test

### Associations with offences and trouble with the police

Individuals with TBI were more likely to have committed at least one offence (unadjusted OR = 1.72, 95% CI 1.32–2.23) and to have been in trouble with the police (unadjusted OR 1.62, 95% CI 1.21–2.17). The association with committing at least one offence was robust to adjustment for pre-birth and childhood confounders, while the association with being in trouble with the police was attenuated. Further adjustment for substance use variables substantially weakened the associations between TBI and offences and TBI and trouble with the police. In the negative control analyses, OI was associated with criminal behaviours (offences: unadjusted OR = 1.48, 95% CI 1.23–1.77; trouble with the police: unadjusted OR = 1.42, 95% CI 1.15–1.74), but while the association with offences was robust to adjustment for pre-birth and childhood confounders and substance use, the association with trouble with the police was attenuated substantially following adjustment. There was no clear evidence for increased odds of either offending or being in trouble with the police in the direct comparison between TBI and OI. These results are shown in Table 3. The findings from the complete case and additional sensitivity analyses did not differ substantially and can be seen in Supplementary Tables S6, S7 and S8.

**Table 3:** Associations between traumatic brain injury and orthopaedic injuries from birth to age 16 years and criminal behaviours at age 17 years

Criminal behaviour	OR (95% CI)			
	Unadjusted	Model 1	Model 2	Model 3
Offences^a,b^				
*n*	3846	3396	2990	2115
TBI vs no injury	1.72 (1.32–2.23)	1.56 (1.17–2.07)	1.67 (1.24–2.24)	1.29 (0.09–1.88)
OI vs no injury	1.48 (1.23–1.77)	1.35 (1.11–1.65)	1.41 (1.14–1.74)	1.67 (1.27–2.19)
TBI vs OI	1.16 (0.87–1.54)	1.15 (0.85–1.56)	1.18 (0.86–1.63)	0.77 (0.52–1.16)
Omnibus *p*	<0.001	0.001	0.001	<0.001
Trouble with the police^a,c^				
*n*	3782	3340	2947	2077
TBI vs no injury	1.62 (1.21–2.17)	1.33 (0.96–1.84)	1.44 (1.03–2.01)	1.17 (0.77–1.77)
OI vs no injury	1.42 (1.15–1.74)	1.21 (0.97–1.52)	1.23 (0.96–1.56)	1.03 (0.75–1.42)
TBI vs OI	1.14 (0.83–1.57)	1.09 (0.77–1.55)	1.17 (0.81–1.69)	1.14 (0.71–1.81)
Omnibus *p*	<0.001	0.064	0.062	0.765

Sample size reduces per adjustment as the participants who are missing covariate data get excluded

*TBI* traumatic brain injury, *OI* orthopaedic injury, *Unadjusted* Injuries from birth to age 16 years with main crime variable in each analysis, *Model 1* As unadjusted with additional adjustment for pre-birth confounders (mother’s age at birth, mother’s education at birth, social class and gender), *Model 2* As Model 1 with additional adjustment for childhood confounders (early life events, parental bonding, positive and negative parenting experiences, maternal alcohol use and maternal tobacco smoking), *Model 3* As Model 2 with additional adjustment for substance use variables

^a^Generalised ordinal regression

^b^Offences measured by self-report questionnaire at age 17 years

^c^Trouble with the police measured by self-report questionnaire at age 17 years

### Associations with conduct problems and peer problems

Participants with TBI were at increased risk of having conduct problems (unadjusted OR = 1.58, 95% CI 1.11–2.25), and this association was slightly strengthened following adjustment for pre-birth and childhood confounders. There was no evidence for an association between TBI status and peer problems. In the negative control analyses, there was no evidence for an association between OI status and conduct or peer problems. There was no clear evidence for increased odds of either conduct or peer problems in the direct comparison between TBI and OI. These results are shown in Table 4. The findings from the complete case and additional sensitivity analyses did not differ substantially and can be seen in Supplementary Tables S9, S10 and S11.

**Table 4:** Associations between traumatic brain injury and orthopaedic injuries from birth to age 16 years and psychiatric symptoms based on the Strengths and Difficulties Questionnaire (SDQ) at age 17 years

SDQ	OR (95% CI)		
	Unadjusted	Model 1	Model 2
Conduct problems^a,b^			
*n*	5634	4997	4493
TBI vs no injury	1.58 (1.11–2.25)	1.78 (1.22–2.59)	1.62 (1.08–2.41)
OI vs no injury	1.15 (0.87–1.50)	1.12 (0.83–1.52)	1.07 (0.78–1.47)
TBI vs OI	1.38 (0.93–2.05)	1.58 (1.03–2.42)	1.51 (0.96–2.37)
Omnibus *p*	0.181	0.242	0.445
Peer problems^a,c^			
*n*	5626	4987	4483
TBI vs no injury	1.11 (0.79–1.55)	0.99 (0.68–1.42)	0.85 (0.57–1.26)
OI vs no injury	0.96 (0.76–1.22)	0.81 (0.62–1.06)	0.79 (0.60–1.05)
TBI vs OI	1.15 (0.79–1.67)	1.21 (0.80–1.83)	1.07 (0.68–1.67)
Omnibus *p*	0.852	0.138	0.090

Sample size reduces per adjustment as the participants who are missing covariate data get excluded

*TBI* traumatic brain injury, *OI* orthopaedic injury, *Unadjusted* Injuries from birth to age 16 years with main SDQ variable in each analysis, *Model 1* As unadjusted with additional adjustment for pre-birth confounders (mother’s age at birth, mother’s education at birth, social class and gender), *Model 2* As Model 1 with additional adjustment for childhood confounders (early life events, parental bonding, positive and negative parenting experiences, maternal alcohol use and maternal tobacco smoking)

^a^Logistic regression

^b^Conduct problems based on parent-completed Strengths and Difficulties Questionnaire at age 17 years

^c^Peer problems based on parent-completed Strengths and Difficulties Questionnaire at age 17 years

### Effects of age at injury: childhood and adolescent injuries

Both childhood (between birth and age 11 years) and adolescent (between age 12 and 16 years) TBI were associated with problematic cannabis use at age 17 years in the unadjusted models (childhood: unadjusted OR = 1.61, 95% CI 1.14–2.28; adjusted OR = 1.45, 95% CI 0.98–2.15; adolescent: unadjusted OR = 1.49, 95% CI 1.11 to 1.99; adjusted OR 1.36, 95% CI 0.98–1.88). Adolescent TBI was also associated with increased hazardous use of alcohol at age 17 years (unadjusted OR = 1.71, 95% CI 1.28–2.27; adjusted OR 1.72, 95% CI 1.25–2.37) and problematic use of tobacco at age 17 years (unadjusted OR = 1.56, 95% CI 1.11–2.19; adjusted OR = 1.71, 95% CI 1.15–2.52). In the negative control analyses, adolescent OI was associated with problematic use of tobacco (unadjusted OR 1.50, 95% CI 1.13–2.00; adjusted OR 1.76, 95% CI 1.25–2.48). There was no evidence of an association between OI status and any of the other substance use measures. Relative to adolescent OI, adolescent TBI was associated with increased odds of alcohol use only (unadjusted OR = 1.61, 95% CI 1.13–2.31; adjusted OR 1.76, 95% CI 1.17–2.63).

The adolescent TBI group were more likely to have committed at least one offence at age 17 years (unadjusted OR = 2.05, 95% CI 1.50–2.80; adjusted OR 1.99, 95% CI 1.40–2.82) and to have been in trouble with the police at age 17 years (unadjusted OR = 1.74, 95% CI 1.22–2.48; adjusted OR = 1.51, 95% CI 1.00–2.29). In negative control analyses, adolescent OI was associated with having committed at least one offence (adolescent: unadjusted OR = 1.89, 95% CI 1.44–2.45; adjusted OR 1.53, 95% CI 1.11–2.11).

Childhood TBI was associated with increased conduct problems on the SDQ at age 17 years (unadjusted OR = 2.20, 95% CI 1.37–3.53; adjusted OR = 1.90, 95% CI 1.11–3.26). As DAWBA information was available at age 15 years, odds ratios were also calculated for the association between childhood TBI and externalising disorders from this scale. DAWBA externalising symptoms are a combination of ODD, CD and ADHD symptoms. The results of the DAWBA analysis can be seen in Supplementary Table S18. Participants with childhood TBI were more likely to have externalising symptoms (unadjusted OR = 2.25, 95% CI 1.32–3.81; adjusted OR = 1.83, 95% CI 0.98–3.41). Analyses of the three separate disorders revealed a strong effect size of TBI on ADHD (adjusted OR = 3.15, 95% CI 1.07–9.28). Relative to childhood OI, childhood TBI was associated with increased odds of conduct problems (unadjusted OR 2.10, 95% CI 1.23–3.57; adjusted OR 1.98, 95% CI 1.08–3.65) and externalising symptoms (unadjusted OR 2.65, 95% CI 1.43–4.91; adjusted 2.11, 95% CI 1.03–4.32). The full sample and complete case analyses for childhood and adolescent injuries are provided in Supplementary Tables S12–S39.

## Discussion

We used data from a longitudinal birth cohort to explore the association between sustaining a mild TBI before age 16 years and subsequent substance use, criminal behaviour and psychiatric symptoms. There are three main findings. First, relative to having no injury, sustaining a mild TBI between birth and age 16 was associated with problematic alcohol, tobacco and cannabis use, a higher likelihood of committing an offence and a higher likelihood of having conduct problems at age 17 years. Second, in negative control analyses, there was evidence that sustaining a mild TBI was associated with hazardous alcohol use relative to sustaining an OI–adding evidence for a possible causal association between TBI and later alcohol misuse—while both mild TBI and OI were associated with committing offences. Third, additional analyses suggest that age at injury may be important for certain outcomes; participants with a mild TBI between birth and age 11 years had higher odds of psychiatric symptoms at age 17 years, while participants who incurred a mild TBI between age 12 and 16 years had higher odds of problematic substance use and criminal behaviours at age 17 years.

The first main finding lends support to results from other birth cohort studies; although the strength of evidence for associations found in our study are somewhat weaker than those in other birth cohorts. This could reflect our use of self-report rather than medical records whereby non-TBI events may be recalled as TBI diluting the true exposure. We found that 7% of the cohort had experienced a TBI by age 16 years, this lies between the rate of 3.8% in the Northern Finland cohort and 31.6% in the CHDS [26]. Mild TBI was associated with 39–67% increased risk of six of the seven outcomes, which is comparable to the 18–52% increased risk of poor adult outcomes reported by Sariaslan and colleagues [9]. The increased odds of higher levels of alcohol consumption amongst the TBI group here is in keeping with the more frequent intoxication reported by 14 year olds with a TBI in The Northern Finland Birth Cohort [10]. In the CHDS, a TBI requiring hospitalisation was associated with increased odds of externalising disorders and substance abuse at age 14 to 16 years [12] and with increased odds of alcohol and drug dependence, and criminal behaviour at age 16 to 25 years [13]. In the current investigation, the association between TBI and criminal offences did not remain once substance use was added as a covariate. McKinlay and colleagues reported a similar association for those injured before age 5 years; however, for those injured from age 6 to 15 years, a strong association remained for arrests and property offences, but not violent offences. They concluded that a certain threshold of TBI may be required for these effects to be seen [13]. However, in our study it was not possible to index the severity of the TBI.

Second, we included a negative control exposure group to increase the confidence that the associations seen between TBI and risk behaviour may be causal, where several previous studies have only used an uninjured control group [27]. We found that participants who had sustained an OI were not at increased risk of problematic substance use or conduct problems compared to the no injury group, providing further support to previous literature. Interestingly, in a direct comparison between the two injury groups, the TBI group was only found to have a higher likelihood of hazardous alcohol use. This finding has implications for the treatment and management of youth post-TBI as alcohol use has previously been linked with recurrent TBI [28] and poorer recovery from TBI [29]. The lack of evidence for an association between TBI and the other risk outcomes when directly compared with OI highlights the importance of exercising caution when drawing conclusions about mild TBI from research that does not take other injuries into account. The association between OI and committing offences was an unexpected finding; one plausible explanation is that there may be common risk factors for both committing crimes and for being involved in accidents that result in physical injury. For example, sensation-seeking has previously been linked with both criminality and spinal cord injuries in a case–control study of 140 male spinal cord injury patients and 140 matched controls [30]. Although both TBI and OI were associated with committing offences, only those with a TBI were more likely to have been in trouble with the police. Previously it has been suggested that having a TBI may be a risk factor for criminal behaviour and it may place an individual at a disadvantage during legal proceedings [31]. Our finding raises the possibility that having a TBI may also be a factor in the initial transition into the legal system. Future studies in prison populations should measure the incidence rate of OI in addition to TBI in order to further explore this relationship.

Third, there were some differences in risk of outcomes for childhood and adolescent TBI. Childhood TBI (aged 0–11 years) was associated with conduct problems, while adolescent TBI (aged 12–16 years) was associated with increased likelihood of problematic alcohol and tobacco use, as well as criminality. Adolescent OI was associated with problematic tobacco use and committing offences, further highlighting a possible role for common risk factors mentioned above. TBI in both age groups showed weak association with cannabis use. Between these age ranges there was a change from parent-reported to self-reported TBI; however, we feel that this change is unlikely to have impacted the findings as it is more appropriate for the offspring to report their own injuries once they have entered secondary education. There may be some differences in severity of the injuries reported from childhood to adolescence—elsewhere the injuries occurring after 15 years were more severe [26]—it would be interesting to assess this possibility in future research.

The prospective birth cohort design is a major strength of this study. Each injury was reported in close proximity to the time it happened, minimising the issue of recall bias. The longitudinal nature of the study allows for causal inference based on the temporal relationship between exposure and outcome. Additionally, the lack of statistical support and weak associations between our negative control group and the main outcomes, with the exception of committing offences, adds to the strength of evidence for a causal association suggested by previous research. On the other hand, the findings from the direct comparison between TBI and OI showing that TBI was only associated with hazardous alcohol use highlights the importance of exercising caution when interpreting findings on mild TBI without inclusion of a negative control group. However, the study is not without limitations. In particular, we were unable to obtain any index of severity based on the TBI measure, meaning that some nuances in effects based on severity may have been missed. For example, increased alcohol use has previously been related to mild but not moderate-to-severe TBI [10]. Nonetheless the items used to identify TBI are similar to existing research, skull fractures based on ICD codes have been used to classify mild TBI elsewhere [10] and self-report questions asking about loss of consciousness have also been utilised [32–34].

Evidence from cross-sectional work suggests that there is a relationship between mild TBI and risk behaviour in youth [4–8]; however, there is a paucity of high quality longitudinal research investigating this association [27]. We have attempted to further explore a potential causal link by using data from a representative birth cohort and including a non-brain related injury group as a negative exposure control. Overall we found that participants who sustained a mild TBI before age 16 years were more likely than those with no injury or with a history of OI to use alcohol to problematic levels at age 17 years. Additionally, sustaining either a mild TBI or OI before age 16 years increased the likelihood of an individual committing offences at age 17 years. The study adds evidence for a possible causal association between mild TBI in youth and later hazardous alcohol use, and highlights the importance of including an extra injury group in mild TBI research.

## Electronic supplementary material

Below is the link to the electronic supplementary material.

Supplementary material 1 (PDF 826 kb)

## References

[1] WHO (2006) Neurological disorders: public health challenges. World Heal Organ. http://www.who.int/mental_health/neurology/neurological_disorders_report_web.pdf. Accessed 14 Oct 2015

[2] Dewan MC, Mummareddy N, Wellons JC, Bonfield CM (2016). The epidemiology of global pediatric traumatic brain injury: a qualitative review. World Neurosurg.

[3] Center for Injury Prevention and Control (2003) Report to congress on mild traumatic brain injury in the United States: steps to prevent a serious public health problem. Atlanta, GA

[4] Ilie G, Mann RE, Hamilton H, Adlaf EM, Boak A, Asbridge M, Rehm J, Cusimano MD (2015). Substance use and related harms among adolescents with and without traumatic brain injury. J Head Trauma Rehabil.

[5] Max JE, Lindgren SD, Knutson C, Pearson CS, Ihrig D, Welborn A (1998). Child and adolescent traumatic brain injury: correlates of disruptive behaviour disorders. Brain Inj.

[6] Ilie G, Mann RE, Boak A, Hamilton HA, Rehm J, Cusimano MD (2016). Possession of weapon and school violence among adolescents and their association with history of traumatic brain injury, substance use and mental health issues. Injury.

[7] Ilie G, Mann RE, Boak A, Adlaf EM, Hamilton H, Asbridge M, Rehm J, Cusimano MD (2014). Suicidality, bullying and other conduct and mental health correlates of traumatic brain injury in adolescents. PLoS One.

[8] Tonks J, Williams WH, Yates P, Slater A (2011). Cognitive correlates of psychosocial outcome following traumatic brain injury in early childhood: comparisons between groups of children aged under and over 10 years of age. Clin Child Psychol Psychiatry.

[9] Sariaslan A, Sharp DJ, Onofrio BMD, Larsson H, Fazel S (2016). Long-term outcomes associated with traumatic brain injury in childhood and adolescence: a nationwide swedish cohort study of a wide range of medical and social outcomes. PLoS Med.

[10] Winqvist S, Jokelainen J, Luukinen H, Hillbom M (2007). Parental alcohol misuse is a powerful predictor for the risk of traumatic brain injury in childhood. Brain Inj.

[11] Timonen M, Miettunen J, Hakko H, Zitting P, Veijola J, Von Wendt L, Räsänen P (2002). The association of preceding traumatic brain injury with mental disorders, alcoholism and criminality: the Northern Finland 1966 Birth Cohort Study. Psychiatry Res.

[12] McKinlay A, Grace R, Horwood J, Fergusson D, MacFarlane M (2009). Adolescent psychiatric symptoms following preschool childhood mild traumatic brain injury: evidence from a birth cohort. J Head Trauma Rehabil.

[13] McKinlay A, Corrigan J, Horwood LJ, Fergusson DM (2014). Substance abuse and criminal activities following traumatic brain injury in childhood, adolescence, and early adulthood. J Head Trauma Rehabil.

[14] Rees PM (2003). Contemporary issues in mild traumatic brain injury. Arch Phys Med Rehabil.

[15] Gage SH, Munafò MR, Smith GD (2016). Causal inference in developmental origins of health and disease (DOHaD) research. Annu Rev Psychol.

[16] Fazel S, Lichtenstein P, Grann M, Langstrom N (2011). Risk of violent crime in individuals with epilepsy and traumatic brain injury: a 35-year swedish population study. PLoS Med.

[17] Boyd A, Golding J, Macleod J, Lawlor DA, Fraser A, Henderson J, Molloy L, Ness A, Ring S, Smith GD (2012). Cohort Profile: the “Children of the 90 s”—the index offspring of the Avon Longitudinal Study of Parents and Children. Int J Epidemiol.

[18] Heatherton TF, Kozlowski LT, Frecker RC, Fagerström KO (1991). The fagerström test for nicotine dependence: a revision of the fagerström tolerance questionnaire. Br J Addict.

[19] Babor TF, Higgins-Biddle JC, Saunders JB, Monteiro MG (2001) The alcohol use disorders identification test. Guidel Prim Care 2:1–40

[20] Legleye S, Piontek D, Kraus L, Morand E, Falissard B (2013). A validation of the cannabis abuse screening test (CAST) using a latent class analysis of the DSM-IV among adolescents. Int J Methods Psychiatr Res.

[21] Cho S, Heron J, Aliev F, Salvatore JE, Lewis G, Macleod J, Hickman M, Maughan B, Kendler KS, Dick DM (2015). Directional relationships between alcohol use and antisocial behavior across adolescence. Alcohol Clin Exp Res.

[22] Goodman R (1997). The strengths and difficulties questionnaire: a research note. J Child Psychol Psychiatry.

[23] Goodman A, Heiervang E, Collishaw S, Goodman R (2011). The “DAWBA bands” as an ordered-categorical measure of child mental health: description and validation in British and Norwegian samples. Soc Psychiatry Psychiatr Epidemiol.

[24] McKinlay A, Kyonka EGE, Grace RC, Horwood LJ, Fergusson DM, MacFarlane MR (2010). An investigation of the pre-injury risk factors associated with children who experience traumatic brain injury. Inj Prev.

[25] Williams R (2006). Generalized ordered logit/partial proportional odds models for ordinal dependent variables. Stata J.

[26] Corrigan JD, Selassie AW, Orman JAL (2010). The epidemiology of traumatic brain injury. J Head Trauma Rehabil.

[27] Kennedy E, Cohen M, Munafò M (2017). Childhood traumatic brain injury and the associations with risk behavior in adolescence and young adulthood: a systematic review. J Head Trauma Rehabil.

[28] Winqvist S, Luukinen H, Jokelainen J, Lehtilahti M, Näyhä S, Hillbom M (2008). Recurrent traumatic brain injury is predicted by the index injury occurring under the influence of alcohol. Brain Inj.

[29] Corrigan JD (1995). Substance abuse as a mediating factor in outcome from traumatic brain injury. Arch Phys Med Rehabil.

[30] Mawson AR, Biundo JJ, Clemmer DI, Jacobs KW, Ktsanes VK, Rice JC (1996). Sensation-seeking, criminality, and spinal cord injury: a case-control study. Am J Epidemiol.

[31] Williams WH, McAuliffe KA, Cohen MH, Parsonage M, Ramsbotham GTLDJ (2015). Traumatic brain injury and juvenile offending: complex causal links offer multiple targets to reduce crime. J Head Trauma Rehabil.

[32] Moore E, Indig D, Haysom L (2014). Traumatic brain injury, mental health, substance use, and offending among incarcerated young people. J Head Trauma Rehabil.

[33] Perron BE, Howard MO (2008). Prevalence and correlates of traumatic brain injury among delinquent youths. Crim Behav Ment Heal.

[34] Williams WH, Cordan G, Mewse AJ, Tonks J, Burgess CNW (2010). Self-reported traumatic brain injury in male young offenders: a risk factor for re-offending, poor mental health and violence?. Neuropsychol Rehabil.

