# Supplementary material for: Substance use, criminal behaviour and psychiatric symptoms following childhood traumatic brain injury: findings from the ALSPAC cohort
Source: Eur Child Adolesc Psychiatry. 2017 Mar 17;26(10):1197–206. doi: 10.1007/s00787-017-0975-1 (PMC5610220; doi:10.1007/s00787-017-0975-1)
Supplement: Supplementary file 1 — Supplementary material 1 (PDF 826 kb) [file 787_2017_975_MOESM1_ESM.pdf]

Supplementary Table 1. Descriptive statistics for participants with injury information included in analyses and participants excluded from analyses due to missing injury information.

	**Included** *(n=11412)*	**Excluded** *(n=4033)*	p value*
	N (%)	N (%)	
**Male**	5849 (51.3)	1786 (51.8)	0.553
**Social class IV – V** [a]	4111 (41.9)	926 (53.6)	<0.001
**Rented subsidised housing**	1208 (11.4)	731 (25.0)	<0.001
**Mother completed secondary school**	6496 (62.1)	1587 (78.1)	<0.001
**Maternal daily smoking**	3034 (28.2)	763 (41.2)	<0.001
**Maternal daily alcohol use**	1408 (13.1)	103 (5.6)	<0.001
**Three or more early life events** [b]	5797 (54.4)	330 (27.1)	<0.001
	M (SD)	M (SD)	
**Maternal age at birth (years)**	28.51 (4.76)	26.16 (5.23)	<0.001
**Bonding at 8 months** [c]	28.23 (3.65)	28.46 (3.87)	0.040
**Positive parenting experience at 21 months** [d]	20.78 (2.74)	20.94 (2.90)	0.145
**Negative parenting experience at 21 months** [d]	5.99 (1.52)	6.00 (1.58)	0.850

Injury from birth to age 16 years (data present n = 11412; data missing n = 4033); ** p values calculated using chi square or analysis of variance;* [a] *highest social class of either parent is skilled non-manual or lower occupation based on the Registrar General's classification of occupations;* [b] *parent-reported questionnaire relating to upsetting events in the child's life completed when offspring was 6, 30, 42 and 81 months old;* [c] *parent-report questionnaire completed when offspring was 8 months old;* [d] *positive and negative parenting experiences based on parent-completed questionnaire when offspring was 21 months old.*

Supplementary Table 2 Descriptive statistics for covariates on complete case sample for all covariates and all substance use (alcohol, tobacco, cannabis) measures.

	**No Injury** (n=1,363)	**TBI** (n=207)	**OI** (n=504)	p value*
	N (%)	N (%)	N (%)	
**Male**	553 (40.6)	112 (54.1)	258 (51.2)	<0.001
**Social Class IV – V** [a]	450 (33.0)	65 (31.4)	168 (33.3)	0.878
**Rented subsidised housing**	69 (5.1)	10 (4.8)	18 (3.6)	0.575
**Mother completed secondary school**	657 (48.2)	93 (44.9)	256 (50.8)	0.338
**Maternal daily smoking**	241 (17.7)	45 (21.7)	92 (18.3)	0.371
**Maternal daily alcohol use**	208 (15.3)	36 (17.4)	73 (14.5)	0.619
**Three or more early life events** [b]	764 (56.0)	132 (63.8)	284 (56.3)	0.120
	M (SD)	M (SD)	M (SD)	
**Maternal age at birth (years)**	29.84 (4.29)	29.42 (4.26)	29.61 (4.49)	0.600
**Bonding at 8 months** [c]	27.93 (3.57)	28.06 (3.20)	27.99 (3.49)	0.844
**Positive parenting experience at 21 months** [d]	5.95 (1.39)	5.94 (1.34)	5.87 (1.34)	0.518
**Negative parenting experience at 21 months** [d]	20.84 (2.67)	20.55 (2.80)	20.90 (2.58)	0.272

TBI: traumatic brain injury; OI: orthopaedic injury; ******* *p values calculated using chi square or analysis of variance;* [a] *highest social class of either parent is skilled non-manual or lower occupation based on the Registrar General's classification of occupations;* [b] *parent-reported questionnaire relating to upsetting events in the child's life completed when offspring was 6, 30, 42 and 81 months old;* [c] *parent-report questionnaire completed when offspring was 8 months old;* [d] *positive and negative parenting experiences based on parent-completed questionnaire when offspring was 21 months old.*

Supplementary Table 3 Associations between traumatic brain injury and orthopaedic injuries from birth to age 16 years and substance use at age 17 years on complete case sample

Substance Use	Unadjusted OR (95% CI)	Model 1 OR (95% CI)	Model 2 OR (95% CI)	Model 3 OR (95% CI)
	Alcohol [a*]			
n	*n = 2074*	*n = 2074*	*n = 2074*	*n = 2074*
TBI vs no Injury	1.54 (1.15 - 2.06)	1.51 (1.13 - 2.03)	1.48 (1.10 - 2.00)	1.31 (0.94 - 1.82)
OI vs no Injury	0.88 (0.72 - 1.09)	0.87 (0.70 - 1.07)	0.87 (0.70 - 1.08)	0.77 (0.61 – 0.98)
TBI vs OI	1.74 (1.26 - 2.41)	1.75 (1.26 - 2.42)	1.71 (1.23 - 2.38)	1.69 (1.17 - 2.45)
Omnibus p	0.541	0.408	0.412	0.080
	Tobacco [b**]			
n	*n = 2074*	*n = 2074*	*n = 2074*	*n = 2074*
TBI vs no Injury	1.36 (0.98 - 1.89)	1.43 (1.02 - 1.99)	1.37 (0.98 - 1.93)	1.09 (0.74 - 1.62)
OI vs no Injury	1.09 (0.85 - 1.39)	1.12 (0.87 - 1.43)	1.13 (0.88 - 1.46)	1.15 (0.86 - 1.55)
TBI vs OI	1.25 (0.86 - 1.81)	1.27 (0.88 - 1.85)	1.21 (0.83 - 1.77)	0.95 (0.61 - 1.47)
Omnibus p	0.341	0.238	0.227	0.331
	Cannabis [c**]			
n	*n = 2074*	*n = 2074*	*n = 2074*	*n = 2074*
TBI vs no Injury	1.60 (1.18 - 2.16)	1.56 (1.15 - 2.11)	1.51 (1.11 - 2.05)	1.23 (0.87 - 1.74)
OI vs no Injury	1.10 (0.88 - 1.37)	1.09 (0.87 - 1.37)	1.09 (0.87 - 1.37)	1.02 (0.79 - 1.33)
TBI vs OI	1.46 (1.04 - 2.03)	1.43 (1.02 - 2.00)	1.39 (0.99 - 1.95)	1.20 (0.82 - 1.77)
Omnibus p	0.200	0.236	0.254	0.718

complete cases had no missing data for the exposure, outcome or covariates. *TBI: traumatic brain injury; OI: orthopaedic injury; *logistic regression; **generalised ordinal regression; [a] alcohol measured using the Alcohol Use Disorder Identification Test (AUDIT); [b] tobacco measured using the Fagerström Test for Nicotine Dependence; [c] cannabis measured using the Cannabis Abuse Screening Test.*

Unadjusted: Injuries from birth to age 16 years with main substance use variable in each analysis
Model 1: As unadjusted with additional adjustment for pre-birth confounders (mother's age at birth, mother's education at birth, social class and gender)
Model 2: As Model 1 with additional adjustment for childhood confounders (early life events, parental bonding, positive and negative parenting experiences, maternal alcohol use and maternal tobacco smoking)
Model 3: As Model 2 with additional adjustment for substance use and crime variables

Supplementary Table 4 Associations between traumatic brain injury from birth to age 16 years, with no additional orthopaedic injury, and substance use at age 17 years compared to orthopaedic injury

Substance Use	Unadjusted<br>OR (95% CI)	Model 1<br>OR (95% CI)	Model 2<br>OR (95% CI)	Model 3<br>OR (95% CI)
	Alcohol [a*]			
n	*n = 3564*	*n = 3148*	*n = 2778*	*n = 1992*
TBI only vs OI	1.48 (1.10 - 1.99)	1.44 (1.05 - 1.98)	1.57 (1.13 - 2.18)	1.98 (1.28 - 3.09)
Omnibus p	0.056	0.313	0.306	0.081
	Tobacco [b**]			
n	*n = 2991*	*n = 2642*	*n = 2326*	*n = 1992*
TBI only vs OI	1.14 (0.78 - 1.66)	1.12 (0.75 - 1.68)	1.04 (0.67 - 1.62)	0.96 (0.56 - 1.63)
Omnibus p	0.094	0.072	0.078	0.374
	Cannabis [c**]			
n	*n = 3843*	*n = 3384*	*n = 2978*	*n = 1992*
TBI only vs OI	1.20 (0.88 - 1.62)	1.09 (0.79 - 1.52)	1.08 (0.76 - 1.53)	1.14 (0.71 - 1.82)
Omnibus p	0.007	0.078	0.109	0.588

Sample size reduces per adjustment as the participants who are missing covariate data get excluded *TBI: traumatic brain injury; OI: orthopaedic injury; *logistic regression; **generalised ordinal regression; [a] alcohol measured using the Alcohol Use Disorder Identification Test (AUDIT); [b] tobacco measured using the Fagerström Test for Nicotine Dependence; [c] cannabis measured using the Cannabis Abuse Screening Test.*

Unadjusted: Injuries from birth to age 16 years with main substance use variable in each analysis
Model 1: As unadjusted with additional adjustment for pre-birth confounders (mother's age at birth, mother's education at birth, social class and gender)
Model 2: As Model 1 with additional adjustment for childhood confounders (early life events, parental bonding, positive and negative parenting experiences, maternal alcohol use and maternal tobacco smoking)
Model 3: As Model 2 with additional adjustment for substance use and crime variables

Supplementary Table 5 Associations between traumatic brain injury from birth to age 16 years, with no additional orthopaedic injury, and substance use at age 17 years compared to orthopaedic injury on complete case sample

Substance Use	Unadjusted<br>OR (95% CI)	Model 1<br>OR (95% CI)	Model 2<br>OR (95% CI)	Model 3<br>OR (95% CI)
	Alcohol [a*]			
n	*n = 1992*	*n = 1992*	*n = 1992*	*n = 1992*
TBI only vs OI	1.81 (1.22 - 2.68)	1.82 (1.22 - 2.70)	1.81 (1.22 - 2.70)	1.98 (1.28 - 3.09)
Omnibus p	0.435	0.328	0.344	0.081
	Tobacco [b**]			
n	*n = 1992*	*n = 1992*	*n = 1992*	*n = 1992*
TBI only vs OI	1.08 (0.68 - 1.71)	1.12 (0.71 - 1.78)	1.10 (0.69 - 1.76)	0.96 (0.56 - 1.63)
Omnibus p	0.446	0.324	0.302	0.374
	Cannabis [c**]			
n	*n = 1992*	*n = 1992*	*n = 1992*	*n = 1992*
TBI only vs OI	1.24 (0.82 - 1.87)	1.23 (0.81 - 1.86)	1.22 (0.80 - 1.85)	1.14 (0.71 - 1.82)
Omnibus p	0.319	0.355	0.365	0.588

complete cases had no missing data for the exposure, outcome or covariates. *TBI: traumatic brain injury; OI: orthopaedic injury; *logistic regression; **generalised ordinal regression; [a] alcohol measured using the Alcohol Use Disorder Identification Test (AUDIT); [b] tobacco measured using the Fagerström Test for Nicotine Dependence; [c] cannabis measured using the Cannabis Abuse Screening Test.*

Unadjusted: Injuries from birth to age 16 years with main substance use variable in each analysis
Model 1: As unadjusted with additional adjustment for pre-birth confounders (mother's age at birth, mother's education at birth, social class and gender)
Model 2: As Model 1 with additional adjustment for childhood confounders (early life events, parental bonding, positive and negative parenting experiences, maternal alcohol use and maternal tobacco smoking)
Model 3: As Model 2 with additional adjustment for substance use and crime variables

Supplementary Table 6 Associations between traumatic brain injury and orthopaedic injuries from birth to age 16 years and criminal behaviours at age 17 years on complete case sample

Criminal Behaviour	Unadjusted OR (95% CI)	Model 1 OR (95% CI)	Model 2 OR (95% CI)	Model 3 OR (95% CI)
	Offences [a]**			
n	*n = 2115*	*n = 2115*	*n = 2115*	*n = 2115*
TBI vs no Injury	1.82 (1.30 - 2.54)	1.62 (1.15 - 2.28)	1.58 (1.13 - 2.23)	1.29 (0.09 - 1.88)
OI vs no Injury	1.71 (1.34 - 2.18)	1.57 (1.22 - 2.01)	1.56 (1.22 - 2.00)	1.67 (1.27 - 2.19)
TBI vs OI	1.06 (0.74 - 1.53)	1.03 (0.72 - 1.49)	1.01 (0.70 - 1.47)	0.77 (0.52 - 1.16)
Omnibus p	<0.001	<0.001	<0.001	<0.001
	Trouble with the Police [b]**			
n	*n = 2077*	*n = 2077*	*n = 2077*	*n = 2077*
TBI vs no Injury	1.73 (1.20 – 2.48)	1.52 (1.05 – 2.21)	1.49 (1.02 – 2.17)	1.17 (0.77 – 1.77)
OI vs no Injury	1.15 (0.87 – 1.53)	1.01 (0.75 – 1.35)	1.02 (0.76 – 1.36)	1.03 (0.75 – 1.42)
TBI vs OI	1.50 (1.00 - 2.25)	1.51 (1.00 - 2.30)	1.46 (0.96 - 2.23)	1.14 (0.71 - 1.81)
Omnibus p	0.158	0.707	0.678	0.765

Complete cases had no missing data for the exposure, outcome or covariates *TBI: traumatic brain injury; OI: orthopaedic injury; **generalised ordinal regression; [a] offences measured by self-report questionnaire at age 17 years; [b] trouble with the police measured by self-report questionnaire at age 17 years.*

Unadjusted: Injuries from birth to age 16 years with main substance use variable in each analysis
Model 1: As unadjusted with additional adjustment for pre-birth confounders (mother's age at birth, mother's education at birth, social class and gender)
Model 2: As Model 1 with additional adjustment for childhood confounders (early life events, parental bonding, positive and negative parenting experiences, maternal alcohol use and maternal tobacco smoking)
Model 3: As Model 2 with additional adjustment for substance use variables

Supplementary Table 7 Associations between traumatic brain injury from birth to age 16 years, with no additional orthopaedic injury, and criminal behaviour at age 17 years compared to orthopaedic injury

Criminal Behaviour	Unadjusted<br>OR (95% CI)	Model 1<br>OR (95% CI)	Model 2<br>OR (95% CI)	Model 3<br>OR (95% CI)
	Offences [a]**			
n	*n = 3719*	*n = 3283*	*n = 2886*	*n = 2031*
TBI only vs OI	1.14 (0.81 - 1.60)	1.15 (0.80 - 1.66)	1.18 (0.81 - 1.73)	0.76 (0.46 - 1.26)
Omnibus p	<0.001	0.001	0.001	<0.001
	Trouble with the Police [b]**			
n	*n = 3657*	*n = 3228*	*n = 2844*	*n = 1995*
TBI only vs OI	0.94 (0.63 - 1.40)	0.86 (0.55 - 1.34)	0.90 (0.57 - 1.44)	0.80 (0.44 - 1.47)
Omnibus p	0.001	0.096	0.102	0.930

Sample size reduces per adjustment as the participants who are missing covariate data get excluded *TBI: traumatic brain injury; OI: orthopaedic injury; **generalised ordinal regression; [a] offences measured by self-report questionnaire at age 17 years; [b] trouble with the police measured by self-report questionnaire at age 17 years.*

Unadjusted: Injuries from birth to age 16 years with main substance use variable in each analysis
Model 1: As unadjusted with additional adjustment for pre-birth confounders (mother's age at birth, mother's education at birth, social class and gender)
Model 2: As Model 1 with additional adjustment for childhood confounders (early life events, parental bonding, positive and negative parenting experiences, maternal alcohol use and maternal tobacco smoking)
Model 3: As Model 2 with additional adjustment for substance use variables

Supplementary Table 8 Associations between traumatic brain injury from birth to age 16 years, with no additional orthopaedic injury, and criminal behaviour at age 17 years compared to orthopaedic injury on complete case sample

Criminal Behaviour	Unadjusted OR (95% CI)	Model 1 OR (95% CI)	Model 2 OR (95% CI)	Model 3 OR (95% CI)
	Offences [a]**			
n	*n = 2031*	*n = 2031*	*n = 2031*	*n = 2031*
TBI only vs OI	0.94 (0.60 - 1.46)	0.95 (0.60 - 1.49)	0.95 (0.60 - 1.50)	0.76 (0.46 - 1.26)
Omnibus p	<0.001	<0.001	<0.001	<0.001
	Trouble with the Police [b]**			
n	*n = 1995*	*n = 1995*	*n = 1995*	*n = 1995*
TBI only vs OI	1.02 (0.60 - 1.74)	1.03 (0.60 - 1.79)	1.02 (0.59 - 1.76)	0.80 (0.44 - 1.47)
Omnibus p	0.299	0.956	0.921	0.930

Complete cases had no missing data for the exposure, outcome or covariates *TBI: traumatic brain injury; OI: orthopaedic injury; **generalised ordinal regression; [a] offences measured by self-report questionnaire at age 17 years; [b] trouble with the police measured by self-report questionnaire at age 17 years.*

Unadjusted: Injuries from birth to age 16 years with main substance use variable in each analysis
Model 1: As unadjusted with additional adjustment for pre-birth confounders (mother's age at birth, mother's education at birth, social class and gender)
Model 2: As Model 1 with additional adjustment for childhood confounders (early life events, parental bonding, positive and negative parenting experiences, maternal alcohol use and maternal tobacco smoking)
Model 3: As Model 2 with additional adjustment for substance use variables

Supplementary Table 9 Associations between traumatic brain injury and orthopaedic injuries from birth to age 16 years and psychiatric symptoms based on the Strengths and Difficulties Questionnaire at age 17 years on complete case sample

SDQ	Unadjusted OR (95% CI)	Model 1 OR (95% CI)	Model 2 OR (95% CI)
	Conduct problems [a*]		
n	*n = 4493*	*n = 4493*	*n = 4493*
TBI vs no Injury	1.64 (1.11 – 2.43)	1.72 (1.16 – 2.55)	1.62 (1.08 – 2.41)
OI vs no Injury	1.08 (0.79 – 1.48)	1.10 (0.80 – 1.50)	1.07 (0.78 – 1.47)
TBI vs OI	1.52 (0.97 - 2.36)	1.57 (1.01 - 2.45)	1.51 (0.96 - 2.37)
Omnibus p	0.391	0.340	0.445
	Peer Problems [b*]		
n	*n = 4483*	*n = 4483*	*n = 4483*
TBI vs no Injury	0.92 (0.62 – 1.37)	0.88 (0.59 – 1.31)	0.85 (0.57 – 1.26)
OI vs no Injury	0.84 (0.64 – 1.11)	0.81 (0.61 – 1.07)	0.79 (0.60 – 1.05)
TBI vs OI	1.10 (0.71 - 1.71)	1.09 (0.70 - 1.69)	1.07 (0.68 - 1.67)
Omnibus p	0.206	0.127	0.090

Complete cases had no missing data for the exposure, outcome or covariates *TBI: traumatic brain injury; OI: orthopaedic injury; *logistic regression; [a] conduct problems based on parent-completed Strengths and Difficulties Questionnaire at age 17 years; [b] peer problems based on parent-completed Strengths and Difficulties Questionnaire at age 17 years.*

Unadjusted: Injuries from birth to age 16 years with main substance use variable in each analysis
Model 1: As unadjusted with additional adjustment for pre-birth confounders (mother's age at birth, mother's education at birth, social class and gender)
Model 2: As Model 1 with additional adjustment for childhood confounders (early life events, parental bonding, positive and negative parenting experiences, maternal alcohol use and maternal tobacco smoking)

Supplementary Table 10 Associations between traumatic brain injury from birth to age 16 years, with no additional orthopaedic injury, and psychiatric symptoms at age 17 years compared to orthopaedic injury

SDQ	Unadjusted<br>OR (95% CI)	Model 1<br>OR (95% CI)	Model 2<br>OR (95% CI)
	Conduct problems [a*]		
n	*n = 2437*	*n = 4818*	*n = 4328*
TBI only vs OI	1.51 (0.96 - 2.38)	1.74 (1.07 - 2.84)	1.80 (1.08 - 3.00)
Omnibus p	0.201	0.285	0.458
	Peer Problems [b*]		
n	*n = 5427*	*n = 4806*	*n = 4316*
TBI only vs OI	1.17 (0.75 - 1.83)	1.17 (0.71 - 1.93)	1.15 (0.68 - 1.96)
Omnibus p	0.829	0.121	0.100

Sample size reduces per adjustment as the participants who are missing covariate data get excluded *TBI: traumatic brain injury; OI: orthopaedic injury; *logistic regression; [a] conduct problems based on parent-completed Strengths and Difficulties Questionnaire at age 17 years; [b] peer problems based on parent-completed Strengths and Difficulties Questionnaire at age 17 years.*

Unadjusted: Injuries from birth to age 16 years with main substance use variable in each analysis
Model 1: As unadjusted with additional adjustment for pre-birth confounders (mother's age at birth, mother's education at birth, social class and gender)
Model 2: As Model 1 with additional adjustment for childhood confounders (early life events, parental bonding, positive and negative parenting experiences, maternal alcohol use and maternal tobacco smoking)

Supplementary Table 11 Associations between traumatic brain injury from birth to age 16 years, with no additional orthopaedic injury, and psychiatric symptoms at age 17 years compared to orthopaedic injury on complete case sample

SDQ	Unadjusted<br>OR (95% CI)	Model 1<br>OR (95% CI)	Model 2<br>OR (95% CI)
	Conduct problems [a*]		
n	*n = 4328*	*n = 4328*	*n = 4328*
TBI only vs OI	1.76 (1.06 - 2.91)	1.85 (1.12 - 3.07)	1.80 (1.08 - 3.00)
Omnibus p	0.412	0.366	0.458
	Peer Problems [b*]		
n	*n = 4316*	*n = 4316*	*n = 4316*
TBI only vs OI	1.15 (0.68 - 1.95)	1.16 (0.68 - 1.96)	1.15 (0.68 - 1.96)
Omnibus p	0.217	0.133	0.100

Complete cases had no missing data for the exposure, outcome or covariates *TBI: traumatic brain injury; OI: orthopaedic injury; *logistic regression;* [a] *conduct problems based on parent-completed Strengths and Difficulties Questionnaire at age 17 years;* [b] *peer problems based on parent-completed Strengths and Difficulties Questionnaire at age 17 years.*

Unadjusted: Injuries from birth to age 16 years with main substance use variable in each analysis
Model 1: As unadjusted with additional adjustment for pre-birth confounders (mother's age at birth, mother's education at birth, social class and gender)
Model 2: As Model 1 with additional adjustment for childhood confounders (early life events, parental bonding, positive and negative parenting experiences, maternal alcohol use and maternal tobacco smoking)

Supplementary Table 12 Association between traumatic brain injury and orthopaedic injuries from birth to age 11 years and substance use at age 17 years

Substance Use	Unadjusted<br>OR (95% CI)	Model 1<br>OR (95% CI)	Model 2<br>OR (95% CI)	Model 3<br>OR (95% CI)
	Alcohol [a*]			
n	*n = 3188*	*n = 2812*	*n = 2381*	*n = 1788*
TBI vs no Injury	1.26 (0.89 - 1.78)	1.28 (0.89 - 1.85)	1.37 (0.93 – 2.02)	1.13 (0.68 - 1.88)
OI vs no Injury	1.16 (0.97 - 1.38)	1.10 (0.91 – 1.33)	1.13 (0.92 - 1.38)	0.81 (0.61 - 1.07)
TBI vs OI	1.09 (0.75 - 1.57)	1.16 (0.78 - 1.72)	1.22 (0.81 - 1.85)	1.40 (0.80 - 2.44)
Omnibus p	0.072	0.231	0.266	0.167
	Tobacco [b**]			
n	*n = 2675*	*n = 2364*	*n = 2084*	*n = 1788*
TBI vs no Injury	1.35 (0.88 – 2.08)	1.31 (0.83 – 2.07)	1.15 (0.69 – 1.92)	1.00 (0.53 - 1.87)
OI vs no Injury	1.01 (0.80 – 1.28)	1.02 (0.79 – 1.32)	1.00 (0.76 – 1.32)	0.83 (0.58 - 1.19)
TBI vs OI	1.34 (0.84 - 2.15)	1.28 (0.78 - 2.12)	1.16 (0.66 - 2.01)	1.20 (0.60 - 2.39)
Omnibus p	0.786	0.739	0.940	0.328
	Cannabis [c**]			
n	*n = 3436*	*n = 3023*	*n = 2668*	*n = 1788*
TBI vs no Injury	1.61 (1.14 - 2.28)	1.44 (0.99 – 2.08)	1.45 (0.98 - 2.15)	1.47 (0.88 - 2.47)
OI vs no Injury	1.17 (0.98 - 1.41)	1.12 (0.92 – 1.37)	1.10 (0.89 - 1.36)	1.03 (0.76 - 1.40)
TBI vs OI	1.38 (0.95 - 2.00)	1.28 (0.86 - 1.91)	1.32 (0.86 - 2.02)	1.43 (0.81 - 2.52)
Omnibus p	0.041	0.162	0.254	0.671

Sample size reduces per adjustment as the participants who are missing covariate data get excluded *TBI: traumatic brain injury; OI: orthopaedic injury; *logistic regression; **generalised ordinal regression; [a] alcohol measured using the Alcohol Use Disorder Identification Test (AUDIT); [b] tobacco measured using the Fagerström Test for Nicotine Dependence; [c] cannabis measured using the Cannabis Abuse Screening Test.*

Unadjusted: Injuries from birth to age 16 years with main substance use variable in each analysis
Model 1: As unadjusted with additional adjustment for pre-birth confounders (mother's age at birth, mother's education at birth, social class and gender)
Model 2: As Model 1 with additional adjustment for childhood confounders (early life events, parental bonding, positive and negative parenting experiences, maternal alcohol use and maternal tobacco smoking)
Model 3: As Model 2 with additional adjustment for substance use and crime variables

Supplementary Table 13 Associations between traumatic brain injury from birth to age 11 years, with no additional orthopaedic injury, and substance use at age 17 years compared to orthopaedic injury

Substance Use	Unadjusted OR (95% CI)	Model 1 OR (95% CI)	Model 2 OR (95% CI)	Model 3 OR (95% CI)
	Alcohol [a*]			
n	*n = 3144*	*n = 2776*	*n = 2454*	*n = 1762*
TBI only vs OI	1.37 (0.89 - 2.11)	1.40 (0.89 - 2.20)	1.47 (0.91 - 2.37)	1.64 (0.87 - 3.13)
Omnibus p	0.059	0.207	0.243	0.181
	Tobacco [b**]			
n	*n = 2640*	*n = 2333*	*n = 2055*	*n = 1762*
TBI only vs OI	1.30 (0.74 - 2.27)	1.31 (0.73 - 2.35)	1.16 (0.61 - 2.21)	0.98 (0.43 - 2.20)
Omnibus p	0.851	0.774	0.968	0.275
	Cannabis [c**]			
n	*n = 3391*	*n = 2986*	*n = 2635*	*n = 1762*
TBI only vs OI	1.53 (1.00 - 2.36)	1.43 (0.91 - 2.25)	1.47 (0.91 - 2.37)	1.51 (0.79 - 2.87)
Omnibus p	0.044	0.164	0.259	0.214

Sample size reduces per adjustment as the participants who are missing covariate data get excluded *TBI: traumatic brain injury; OI: orthopaedic injury; *logistic regression; **generalised ordinal regression; [a] alcohol measured using the Alcohol Use Disorder Identification Test (AUDIT); [b] tobacco measured using the Fagerström Test for Nicotine Dependence; [c] cannabis measured using the Cannabis Abuse Screening Test.*

Unadjusted: Injuries from birth to age 16 years with main substance use variable in each analysis
Model 1: As unadjusted with additional adjustment for pre-birth confounders (mother's age at birth, mother's education at birth, social class and gender)
Model 2: As Model 1 with additional adjustment for childhood confounders (early life events, parental bonding, positive and negative parenting experiences, maternal alcohol use and maternal tobacco smoking)
Model 3: As Model 2 with additional adjustment for substance use and crime variables

Supplementary Table 14 Associations between traumatic brain injury and orthopaedic injuries from birth to age 11 years and criminal behaviour at age 17 years

Criminal Behaviour	Unadjusted<br>OR (95% CI)	Model 1<br>OR (95% CI)	Model 2<br>OR (95% CI)	Model 3<br>OR (95% CI)
	Offences [a]**			
n	*n = 3325*	*n = 2931*	*n = 2584*	*n = 1818*
TBI vs no Injury	1.26 (0.82 - 1.94)	1.15 (0.72 - 1.83)	1.25 (0.77 - 2.02)	0.97 (0.53 - 1.79)
OI vs no Injury	1.30 (1.05 - 1.62)	1.31 (1.03 - 1.66)	1.37 (1.06 - 1.75)	1.78 (1.30 - 2.45)
TBI vs OI	0.97 (0.61 - 1.53)	0.88 (0.53 - 1.44)	0.91 (0.54 - 1.53)	0.54 (0.28 - 1.04)
Omnibus p	0.013	0.025	0.012	0.001
	Trouble with the Police [b]**			
n	*n = 3275*	*n = 2886*	*n = 2549*	*n = 1790*
TBI vs no Injury	1.44 (0.90 – 2.28)	1.20 (0.73 – 1.98)	1.31 (0.78 - 2.21)	1.17 (0.62 - 2.22)
OI vs no Injury	1.34 (1.05 – 1.70)	1.28 (0.99 - 1.67)	1.27 (0.96 - 1.69)	1.15 (0.79 - 1.69)
TBI vs OI	1.06 (0.66 - 1.76)	0.94 (0.55 - 1.60)	1.03 (0.59 - 1.81)	1.02 (0.50 - 2.06)
Omnibus p	0.012	0.056	0.078	0.432

Sample size reduces per adjustment as the participants who are missing covariate data get excluded. *TBI: traumatic brain injury; OI: orthopaedic injury; **generalised ordinal regression; [a] offences measured by self-report questionnaire at age 17 years; [b] trouble with the police measured by self-report questionnaire at age 17 years.*

Unadjusted: Injuries from birth to age 16 years with main substance use variable in each analysis
Model 1: As unadjusted with additional adjustment for pre-birth confounders (mother's age at birth, mother's education at birth, social class and gender)
Model 2: As Model 1 with additional adjustment for childhood confounders (early life events, parental bonding, positive and negative parenting experiences, maternal alcohol use and maternal tobacco smoking)
Model 3: As Model 2 with additional adjustment for substance use variables

Supplementary Table 15 Associations between traumatic brain injury from birth to age 11 years, with no additional orthopaedic injury, and criminal behaviour at age 17 years compared to orthopaedic injury

Criminal Behaviour	Unadjusted OR (95% CI)	Model 1 OR (95% CI)	Model 2 OR (95% CI)	Model 3 OR (95% CI)
	Offences [a]**			
n	*n = 3285*	*n = 2898*	*n = 2555*	*n = 1792*
TBI only vs OI	1.08 (0.64 - 1.83)	1.00 (0.58 - 1.75)	1.07 (0.60 - 1.91)	0.65 (0.31 - 1.34)
Omnibus p	0.012	0.021	0.010	<0.001
	Trouble with the Police [b]**			
n	*n = 3235*	*n = 2852*	*n = 2519*	*n = 1764*
TBI only vs OI	1.14 (0.65 - 2.00)	0.98 (0.53 - 1.79)	1.08 (0.57 - 2.05)	1.04 (0.47 - 2.29)
Omnibus p	0.012	0.055	0.080	0.434

Sample size reduces per adjustment as the participants who are missing covariate data get excluded. *TBI: traumatic brain injury; OI: orthopaedic injury; **generalised ordinal regression; [a] offences measured by self-report questionnaire at age 17 years; [b] trouble with the police measured by self-report questionnaire at age 17 years.*

Unadjusted: Injuries from birth to age 16 years with main substance use variable in each analysis
Model 1: As unadjusted with additional adjustment for pre-birth confounders (mother's age at birth, mother's education at birth, social class and gender)
Model 2: As Model 1 with additional adjustment for childhood confounders (early life events, parental bonding, positive and negative parenting experiences, maternal alcohol use and maternal tobacco smoking)
Model 3: As Model 2 with additional adjustment for substance use variables

Supplementary Table 16 Associations between traumatic brain injury and orthopaedic injuries from birth to age 11 years and psychiatric symptoms based on the Strengths and Difficulties Questionnaire at age 17 years

SDQ	Unadjusted<br>OR (95% CI)	Model 1<br>OR (95% CI)	Model 2<br>OR (95% CI)
	Conduct problems [a*]		
n	*n = 4923*	*n = 4372*	*n = 3937*
TBI vs no Injury	2.20 (1.37 – 3.53)	2.33 (1.41 – 3.85)	1.90 (1.11 - 3.26)
OI vs no Injury	1.05 (0.76 – 1.44)	0.99 (0.69 – 1.41)	0.96 (0.66 - 1.39)
TBI vs OI	2.10 (1.23 - 3.57)	2.35 (1.33 - 4.17)	1.98 (1.08 - 3.65)
Omnibus p	0.442	0.656	0.884
	Peer Problems [b*]		
n	*n = 4912*	*n = 4359*	*n = 3924*
TBI vs no Injury	1.51 (0.95 – 2.39)	1.30 (0.79 – 2.13)	0.99 (0.57 - 1.21)
OI vs no Injury	1.01 (0.77 – 1.31)	0.91 (0.68 – 1.22)	0.89 (0.65 - 1.21)
TBI vs OI	1.51 (0.91 - 2.49)	1.42 (0.83 - 2.45)	1.12 (0.61 - 2.05)
Omnibus p	0.780	0.666	0.456

Sample size reduces per adjustment as the participants who are missing covariate data get excluded. *TBI: traumatic brain injury; OI: orthopaedic injury; *logistic regression; [a] conduct problems based on parent-completed Strengths and Difficulties Questionnaire at age 17 years; [b] peer problems based on parent-completed Strengths and Difficulties Questionnaire at age 17 years.*

Unadjusted: Injuries from birth to age 16 years with main substance use variable in each analysis
Model 1: As unadjusted with additional adjustment for pre-birth confounders (mother's age at birth, mother's education at birth, social class and gender)
Model 2: As Model 1 with additional adjustment for childhood confounders (early life events, parental bonding, positive and negative parenting experiences, maternal alcohol use and maternal tobacco smoking)

Supplementary Table 17 Associations between traumatic brain injury from birth to age 11 years, with no additional orthopaedic injury, and psychiatric symptoms at age 17 years compared to orthopaedic injury

SDQ	Unadjusted<br>OR (95% CI)	Model 1<br>OR (95% CI)	Model 2<br>OR (95% CI)
	Conduct problems [a*]		
n	*n = 4854*	*n = 4310*	*n = 3880*
TBI only vs OI	2.65 (1.48 - 4.74)	3.04 (1.64 - 5.64)	2.77 (1.45 - 5.31)
Omnibus p	0.450	0.651	0.826
	Peer Problems [b*]		
n	*n = 4842*	*n = 4296*	*n = 3866*
TBI only vs OI	1.58 (0.88 - 2.84)	1.47 (0.78 - 2.77)	1.28 (0.64 - 2.54)
Omnibus p	0.828	0.639	0.481

Sample size reduces per adjustment as the participants who are missing covariate data get excluded. *TBI: traumatic brain injury; OI: orthopaedic injury; *logistic regression; [a] conduct problems based on parent-completed Strengths and Difficulties Questionnaire at age 17 years; [b] peer problems based on parent-completed Strengths and Difficulties Questionnaire at age 17 years.*

Unadjusted: Injuries from birth to age 16 years with main substance use variable in each analysis
Model 1: As unadjusted with additional adjustment for pre-birth confounders (mother's age at birth, mother's education at birth, social class and gender)
Model 2: As Model 1 with additional adjustment for childhood confounders (early life events, parental bonding, positive and negative parenting experiences, maternal alcohol use and maternal tobacco smoking)

Supplementary Table 18 Associations between traumatic brain injury and orthopaedic injuries from birth to age 11 years and psychiatric symptoms based on the Development and Well-Being Assessment (DAWBA) at age 15 years

DAWBA	Unadjusted OR (95% CI)	Model 1 OR (95% CI)	Model 2 OR (95% CI)
	Externalising Behaviour [a*]		
n	*n = 3994*	*n = 3515*	*n = 3112*
TBI vs no Injury	2.25 (1.32 – 3.81)	2.35 (1.35 – 4.11)	1.83 (0.98 - 3.41)
OI vs no Injury	0.85 (0.57 – 1.27)	0.90 (0.59 – 1.39)	0.87 (0.55 - 1.38)
TBI vs OI	2.65 (1.43 - 4.91)	2.61 (1.36 - 4.98)	2.11 (1.03 - 4.32)
Omnibus p	0.779	0.970	0.796
	ODD [b**]		
n	*n = 3983*	*n = 3506*	*n = 3105*
TBI vs no Injury	2.28 (1.28 – 4.07)	2.42 (1.31 – 4.55)	1.78 (0.89 - 3.58)
OI vs no Injury	0.81 (0.51 – 1.28)	0.98 (0.61 – 1.56)	0.98 (0.60 - 1.61)
TBI vs OI	2.82 (1.42 - 5.59)	2.48 (1.22 - 5.02)	1.82 (0.82 - 4.01)
Omnibus p	0.673	0.730	0.851
	CD [c**]		
n	*n = 3982*	*n = 3505*	*n = 3104*
TBI vs no Injury	1.09 (0.34 – 3.55)	0.83 (0.20 – 3.53)	0.72 (0.17 - 3.10)
OI vs no Injury	0.66 (0.31 - 1.40)	0.78 (0.36 – 1.70)	0.85 (0.39 - 1.86)
TBI vs OI	1.66 (0.44 - 6.33)	1.06 (0.22 - 5.10)	0.94 (0.17 - 4.13)
Omnibus p	0.301	0.523	0.636
	ADHD [d**]		
n	*n = 3994*	*n = 3515*	*n = 3112*
TBI vs no Injury	3.15 (1.07 – 9.28)	2.86 (0.94 – 8.70)	3.02 (0.97 - 9.36)
OI vs no Injury	1.42 (0.62 - 3.21)	0.91 (0.34 – 2.47)	0.57 (0.16 - 1.98)
TBI vs OI	2.23 (0.66 - 7.48)	3.14 (0.82 - 12.02)	5.28 (1.14 - 24.51)
Omnibus p	0.274	0.891	0.623

Sample size reduces per adjustment as the participants who are missing covariate data get excluded. *TBI: traumatic brain injury; OI: orthopaedic injury;*
** logistic regression; [a] externalising disorder symptoms based on the Development and Well-being Assessment (DAWBA) self-reported at age 15 years;*

[b] *ODD: oppositional defiant disorder based on the Development and Well-being Assessment (DAWBA) self-reported at age 15 years;* [c] *CD: conduct disorder based on the Development and Well-being Assessment (DAWBA) self-reported at age 15 years;* [d] *ADHD: attentional defiant hyperactivity disorder based on the Development and Well-being Assessment (DAWBA) self-reported at age 15 years*

Unadjusted: Injuries from birth to age 16 years with main substance use variable in each analysis
Model 1: As unadjusted with additional adjustment for pre-birth confounders (mother's age at birth, mother's education at birth, social class and gender)
Model 2: As Model 1 with additional adjustment for childhood confounders (early life events, parental bonding, positive and negative parenting experiences, maternal alcohol use and maternal tobacco smoking)

Table 19 Associations between traumatic brain injury from birth to age 11 years, with no additional orthopaedic injury, and psychiatric symptoms based on the Development and Well-Being Assessment (DAWBA) at age 15 years compared to orthopaedic injury

DAWBA	Unadjusted<br>OR (95% CI)	Model 1<br>OR (95% CI)	Model 2<br>OR (95% CI)
	Externalising Behaviour [a*]		
n	*n = 3931*	*n = 3460*	*n = 3063*
TBI only vs OI	2.34 (1.11 - 4.92)	2.20 (1.00 - 4.83)	1.99 (0.85 - 4.64)
Omnibus p	0.609	0.833	0.698
	ODD [b**]		
n	*n = 3920*	*n = 3451*	*n = 3056*
TBI only vs OI	2.76 (1.25 - 6.12)	2.29 (0.99 - 5.28)	1.94 (0.79 - 4.79)
Omnibus p	0.553	0.876	0.901
	CD [c**]		
n	*n = 3919*	*n = 3450*	*n = 3055*
TBI only vs OI	1.69 (0.35 - 8.06)	0.83 (0.10 - 6.77)	0.67 (0.08 - 5.60)
Omnibus p	0.294	0.512	0.652
	ADHD		
n	*n = 3931*	*n = 3460*	*n = 3063*
TBI only vs OI	1.68 (0.35 - 8.03)	2.53 (0.48 - 13.39)	4.03 (0.65 - 25.14)
Omnibus p	0.345	0.990	0.514

Sample size reduces per adjustment as the participants who are missing covariate data get excluded. *TBI: traumatic brain injury; OI: orthopaedic injury; * logistic regression; [a] externalising disorder symptoms based on the Development and Well-being Assessment (DAWBA) self-reported at age 15 years; [b] ODD: oppositional defiant disorder based on the Development and Well-being Assessment (DAWBA) self-reported at age 15 years; [c] CD: conduct disorder based on the Development and Well-being Assessment (DAWBA) self-reported at age 15 years; [d] ADHD: attentional defiant hyperactivity disorder based on the Development and Well-being Assessment (DAWBA) self-reported at age 15 years*

Unadjusted: Injuries from birth to age 16 years with main substance use variable in each analysis
Model 1: As unadjusted with additional adjustment for pre-birth confounders (mother's age at birth, mother's education at birth, social class and gender)
Model 2: As Model 1 with additional adjustment for childhood confounders (early life events, parental bonding, positive and negative parenting experiences, maternal alcohol use and maternal tobacco smoking)

Supplementary Table 20 Association between traumatic brain injury and orthopaedic injuries from birth to age 11 years and substance use at age 17 years on complete case sample

Substance Use	Unadjusted OR (95% CI)	Model 1 OR (95% CI)	Model 2 OR (95% CI)	Model 3 OR (95% CI)
	Alcohol [a]*			
n	*n = 1788*	*n = 1788*	*n = 1788*	*n = 1788*
TBI vs no Injury	1.25 (0.80 - 1.96)	1.24 (0.79 - 1.94)	1.18 (0.75 - 1.86)	1.13 (0.68 - 1.88)
OI vs no Injury	0.88 (0.69 - 1.13)	0.88 (0.69 - 1.12)	0.87 (0.68 - 1.12)	0.81 (0.61 - 1.07)
TBI vs OI	1.42 (0.87 - 2.31)	1.41 (0.86 - 2.30)	1.35 (0.82 - 2.21)	1.40 (0.80 - 2.44)
Omnibus p	0.429	0.388	0.360	0.167
	Tobacco [b]**			
n	*n = 1788*	*n = 1788*	*n = 1788*	*n = 1788*
TBI vs no Injury	1.09 (0.64 - 1.87)	1.08 (0.63 - 1.85)	0.99 (0.57 - 1.72)	1.00 (0.53 - 1.87)
OI vs no Injury	0.90 (0.66 - 1.21)	0.91 (0.68 - 1.24)	0.91 (0.67 - 1.23)	0.83 (0.58 - 1.19)
TBI vs OI	1.22 (0.68 - 2.19)	1.18 (0.65 - 2.13)	1.09 (0.60 - 2.00)	1.20 (0.60 - 2.39)
Omnibus p	0.518	0.596	0.532	0.328
	Cannabis [c]**			
n	*n = 1788*	*n = 1788*	*n = 1788*	*n = 1788*
TBI vs no Injury	1.45 (0.92 - 2.29)	1.47 (0.92 - 2.32)	1.37 (0.86 - 2.18)	1.47 (0.88 - 2.47)
OI vs no Injury	1.03 (0.79 - 1.33)	1.05 (0.80 - 1.36)	1.04 (0.80 - 1.36)	1.03 (0.76 - 1.40)
TBI vs OI	1.41 (0.86 - 2.33)	1.40 (0.85 - 2.32)	1.32 (0.79 - 2.19)	1.43 (0.81 - 2.52)
Omnibus p	0.659	0.557	0.615	0.671

Complete cases had no missing data for the exposure, outcome or covariates. *TBI: traumatic brain injury; OI: orthopaedic injury; *logistic regression; **generalised ordinal regression; [a] alcohol measured using the Alcohol Use Disorder Identification Test (AUDIT); [b] tobacco measured using the Fagerström Test for Nicotine Dependence; [c] cannabis measured using the Cannabis Abuse Screening Test.*

Unadjusted: Injuries from birth to age 16 years with main substance use variable in each analysis
Model 1: As unadjusted with additional adjustment for pre-birth confounders (mother's age at birth, mother's education at birth, social class and gender)
Model 2: As Model 1 with additional adjustment for childhood confounders (early life events, parental bonding, positive and negative parenting experiences, maternal alcohol use and maternal tobacco smoking)
Model 3: As Model 2 with additional adjustment for substance use and crime variables

Supplementary Table 21 Associations between traumatic brain injury from birth to age 11 years, with no additional orthopaedic injury, and substance use at age 17 years compared to orthopaedic injury on complete case sample

Substance Use	Unadjusted OR (95% CI)	Model 1 OR (95% CI)	Model 2 OR (95% CI)	Model 3 OR (95% CI)
	Alcohol [a]*			
n	*n = 1762*	*n = 1762*	*n = 1762*	*n = 1762*
TBI only vs OI	1.72 (0.98 - 3.04)	1.70 (0.96 - 3.02)	1.61 (0.91 - 2.87)	1.64 (0.87 - 3.13)
Omnibus p	0.453	0.412	0.383	0.181
	Tobacco [b]**			
n	*n = 1762*	*n = 1762*	*n = 1762*	*n = 1762*
TBI only vs OI	1.18 (0.59 - 2.35)	1.17 (0.58 - 2.34)	1.07 (0.53 - 2.17)	0.98 (0.43 - 2.20)
Omnibus p	0.495	0.577	0.511	0.275
	Cannabis [c]**			
n	*n = 1762*	*n = 1762*	*n = 1762*	*n = 1762*
TBI only vs OI	1.58 (0.89 - 2.81)	1.58 (0.88 - 2.81)	1.47 (0.82 - 2.63)	1.51 (0.79 - 2.87)
Omnibus p	0.672	0.568	0.622	0.214

Complete cases had no missing data for the exposure, outcome or covariates. *TBI: traumatic brain injury; OI: orthopaedic injury; *logistic regression; **generalised ordinal regression; [a] alcohol measured using the Alcohol Use Disorder Identification Test (AUDIT); [b] tobacco measured using the Fagerström Test for Nicotine Dependence; [c] cannabis measured using the Cannabis Abuse Screening Test.*

Unadjusted: Injuries from birth to age 16 years with main substance use variable in each analysis
Model 1: As unadjusted with additional adjustment for pre-birth confounders (mother's age at birth, mother's education at birth, social class and gender)
Model 2: As Model 1 with additional adjustment for childhood confounders (early life events, parental bonding, positive and negative parenting experiences, maternal alcohol use and maternal tobacco smoking)
Model 3: As Model 2 with additional adjustment for substance use and crime variables

Supplementary Table 22 Associations between traumatic brain injury and orthopaedic injuries from birth to age 11 years and criminal behaviour at age 17 years on complete case sample

Criminal Behaviour	Unadjusted<br>OR (95% CI)	Model 1<br>OR (95% CI)	Model 2<br>OR (95% CI)	Model 3<br>OR (95% CI)
	Offences [a]**			
n	*n = 1818*	*n = 1818*	*n = 1818*	*n = 1818*
TBI vs no Injury	1.28 (0.74 - 2.21)	1.12 (0.64 - 1.96)	1.06 (0.60 - 1.86)	0.97 (0.53 - 1.79)
OI vs no Injury	1.57 (1.18 - 2.08)	1.57 (1.18 - 2.10)	1.57 (1.17 - 2.09)	1.78 (1.30 - 2.45)
TBI vs OI	0.82 (0.46 - 1.46)	0.71 (0.39 - 1.29)	0.68 (0.37 - 1.23)	0.54 (0.28 - 1.04)
Omnibus p	0.002	0.002	0.003	0.001
	Trouble with the Police [b]**			
n	*n = 1790*	*n = 1790*	*n = 1790*	*n = 1790*
TBI vs no Injury	1.50 (0.85 - 2.63)	1.24 (0.69 - 2.22)	1.17 (0.65 - 2.11)	1.17 (0.62 - 2.22)
OI vs no Injury	1.11 (0.79 - 1.54)	1.06 (0.75 - 1.50)	1.07 (0.76 - 1.51)	1.15 (0.79 - 1.69)
TBI vs OI	1.35 (0.73 - 2.51)	1.16 (0.61 - 2.21)	1.09 (0.57 - 2.08)	1.02 (0.50 - 2.06)
Omnibus p	0.422	0.654	0.635	0.432

Complete cases had no missing data for the exposure, outcome or covariates. *TBI: traumatic brain injury; OI: orthopaedic injury; **generalised ordinal regression; [a] offences measured by self-report questionnaire at age 17 years; [b] trouble with the police measured by self-report questionnaire at age 17 years.*

Unadjusted: Injuries from birth to age 16 years with main substance use variable in each analysis
Model 1: As unadjusted with additional adjustment for pre-birth confounders (mother's age at birth, mother's education at birth, social class and gender)
Model 2: As Model 1 with additional adjustment for childhood confounders (early life events, parental bonding, positive and negative parenting experiences, maternal alcohol use and maternal tobacco smoking)
Model 3: As Model 2 with additional adjustment for substance use variables

Supplementary Table 23 Associations between traumatic brain injury from birth to age 11 years, with no additional orthopaedic injury, and criminal behaviour at age 17 years compared to orthopaedic injury on complete case sample

Criminal Behaviour	Unadjusted<br>OR (95% CI)	Model 1<br>OR (95% CI)	Model 2<br>OR (95% CI)	Model 3<br>OR (95% CI)
	Offences [a]**			
n	*n = 1792*	*n = 1792*	*n = 1792*	*n = 1792*
TBI only vs OI	0.95 (0.49 - 1.84)	0.83 (0.42 - 1.62)	0.80 (0.40 - 1.57)	0.65 (0.31 - 1.34)
Omnibus p	0.001	0.002	0.002	<0.001
	Trouble with the Police [b]**			
n	*n = 1764*	*n = 1764*	*n = 1764*	*n = 1764*
TBI only vs OI	1.48 (0.74 - 2.99)	1.25 (0.61 - 2.58)	1.16 (0.56 - 2.42)	1.04 (0.47 - 2.29)
Omnibus p	0.431	0.650	0.634	0.434

Complete cases had no missing data for the exposure, outcome or covariates. *TBI: traumatic brain injury; OI: orthopaedic injury; **generalised ordinal regression; [a] offences measured by self-report questionnaire at age 17 years; [b] trouble with the police measured by self-report questionnaire at age 17 years.*

Unadjusted: Injuries from birth to age 16 years with main substance use variable in each analysis
Model 1: As unadjusted with additional adjustment for pre-birth confounders (mother's age at birth, mother's education at birth, social class and gender)
Model 2: As Model 1 with additional adjustment for childhood confounders (early life events, parental bonding, positive and negative parenting experiences, maternal alcohol use and maternal tobacco smoking)
Model 3: As Model 2 with additional adjustment for substance use variables

Supplementary Table 24 Associations between traumatic brain injury and orthopaedic injuries from birth to age 11 years and psychiatric symptoms based on the Strengths and Difficulties Questionnaire at age 17 years on complete case sample

SDQ	Unadjusted<br>OR (95% CI)	Model 1<br>OR (95% CI)	Model 2<br>OR (95% CI)
	Conduct problems [a*]		
n	*n = 3937*	*n = 3937*	*n = 3937*
TBI vs no Injury	2.14 (1.26 – 3.63)	2.17 (1.28 - 3.70)	1.90 (1.11 - 3.26)
OI vs no Injury	0.98 (0.68 – 1.41)	0.99 (0.68 – 1.43)	0.96 (0.66 - 1.39)
TBI vs OI	2.19 (1.20 - 3.98)	2.20 (1.21 - 4.01)	1.98 (1.08 - 3.65)
Omnibus p	0.752	0.708	0.884
	Peer Problems [b*]		
n	*n = 3924*	*n = 3924*	*n = 3924*
TBI vs no Injury	1.17 (0.68 - 2.02)	1.10 (0.63 - 1.90)	0.99 (0.57 - 1.21)
OI vs no Injury	0.92 (0.68 - 1.25)	0.91 (0.67 - 1.24)	0.89 (0.65 - 1.21)
TBI vs OI	1.26 (0.70 - 2.29)	1.20 (0.66 - 2.19)	1.12 (0.61 - 2.05)
Omnibus p	0.684	0.605	0.456

Complete cases had no missing data for the exposure, outcome or covariates. *TBI: traumatic brain injury; OI: orthopaedic injury; *logistic regression; [a] conduct problems based on parent-completed Strengths and Difficulties Questionnaire at age 17 years; [b] peer problems based on parent-completed Strengths and Difficulties Questionnaire at age 17 years.*

Unadjusted: Injuries from birth to age 16 years with main substance use variable in each analysis
Model 1: As unadjusted with additional adjustment for pre-birth confounders (mother's age at birth, mother's education at birth, social class and gender)
Model 2: As Model 1 with additional adjustment for childhood confounders (early life events, parental bonding, positive and negative parenting experiences, maternal alcohol use and maternal tobacco smoking)

Supplementary Table 25 Associations between traumatic brain injury from birth to age 11 years, with no additional orthopaedic injury, and psychiatric symptoms at age 17 years compared to orthopaedic injury on complete case sample

SDQ	Unadjusted OR (95% CI)	Model 1 OR (95% CI)	Model 2 OR (95% CI)
	Conduct problems [a]*		
n	*n = 3880*	*n = 3880*	*n = 3880*
TBI only vs OI	3.02 (1.60 - 5.69)	3.05 (1.61 - 5.76)	2.77 (1.45 - 5.31)
Omnibus p	0.716	0.667	0.826
	Peer Problems [b]*		
n	*n = 3866*	*n = 3866*	*n = 3866*
TBI only vs OI	1.42 (0.72 - 2.80)	1.36 (0.69 - 2.69)	1.28 (0.64 - 2.54)
Omnibus p	0.695	0.623	0.481

Complete cases had no missing data for the exposure, outcome or covariates. *TBI: traumatic brain injury; OI: orthopaedic injury; *logistic regression; [a] conduct problems based on parent-completed Strengths and Difficulties Questionnaire at age 17 years; [b] peer problems based on parent-completed Strengths and Difficulties Questionnaire at age 17 years.*

Unadjusted: Injuries from birth to age 16 years with main substance use variable in each analysis
Model 1: As unadjusted with additional adjustment for pre-birth confounders (mother's age at birth, mother's education at birth, social class and gender)
Model 2: As Model 1 with additional adjustment for childhood confounders (early life events, parental bonding, positive and negative parenting experiences, maternal alcohol use and maternal tobacco smoking)

Supplementary Table 26 Associations between traumatic brain injury and orthopaedic injuries from birth to age 11 years and psychiatric symptoms based on the Development and Well-Being Assessment (DAWBA) at age 15 years on complete case sample

DAWBA	Unadjusted<br>OR (95% CI)	Model 1<br>OR (95% CI)	Model 2<br>OR (95% CI)
	Externalising Behaviour [a*]		
n	*n = 3112*	*n = 3112*	*n = 3112*
TBI vs no Injury	2.19 (1.20 - 4.00)	2.11 (1.15 - 3.88)	1.83 (0.98 - 3.41)
OI vs no Injury	0.88 (0.56 - 1.39)	0.88 (0.55 - 1.38)	0.87 (0.55 - 1.38)
TBI vs OI	2.49 (1.24 - 5.01)	2.41 (1.19 - 4.87)	2.11 (1.03 - 4.32)
Omnibus p	0.894	0.866	0.796
	ODD [b**]		
n	*n = 3105*	*n = 3105*	*n = 3105*
TBI vs no Injury	2.11 (1.07 - 4.17)	2.04 (1.03 - 4.04)	1.78 (0.89 - 3.58)
OI vs no Injury	0.98 (0.60 - 1.60)	0.98 (0.60 - 1.60)	0.98 (0.60 - 1.61)
TBI vs OI	2.15 (0.99 - 4.67)	2.08 (0.96 - 4.54)	1.82 (0.82 - 4.01)
Omnibus p	0.797	0.820	0.851
	CD [c**]		
n	*n = 3104*	*n = 3104*	*n = 3104*
TBI vs no Injury	0.91 (0.22 – 3.82)	0.90 (0.21 – 3.80)	0.72 (0.17 - 3.10)
OI vs no Injury	0.83 (0.38 - 1.81)	0.83 (0.38 - 1.81)	0.85 (0.39 - 1.86)
TBI vs OI	1.09 (0.23 - 5.19)	1.08 (0.23 - 5.17)	0.94 (0.17 - 4.13)
Omnibus p	0.641	0.636	0.636
	ADHD [d**]		
n	*n = 3112*	*n = 3112*	*n = 3112*
TBI vs no Injury	3.69 (1.23 - 11.12)	3.39 (1.11 - 10.37)	3.02 (0.97 - 9.36)
OI vs no Injury	0.62 (0.18 - 2.13)	0.60 (0.18 - 2.07)	0.57 (0.16 - 1.98)
TBI vs OI	5.94 (1.31 - 26.81)	5.62 (1.23 - 25.65)	5.28 (1.14 - 24.51)
Omnibus p	0.758	0.702	0.623

Complete cases had no missing data for the exposure, outcome or covariates. *TBI: traumatic brain injury; OI: orthopaedic injury; * logistic regression; [a] externalising disorder symptoms based on the Development and Well-being Assessment (DAWBA) self-reported at age 15 years; [b] ODD: oppositional defiant disorder based on the Development and Well-being Assessment (DAWBA) self-reported at age 15 years; [c] CD: conduct disorder based on the Development and Well-being Assessment (DAWBA) self-reported at age 15 years; [d] ADHD: attentional defiant hyperactivity disorder based on the Development and Well-being Assessment (DAWBA) self-reported at age 15 years*

Unadjusted: Injuries from birth to age 16 years with main substance use variable in each analysis
Model 1: As unadjusted with additional adjustment for pre-birth confounders (mother's age at birth, mother's education at birth, social class and gender)
Model 2: As Model 1 with additional adjustment for childhood confounders (early life events, parental bonding, positive and negative parenting experiences, maternal alcohol use and maternal tobacco smoking)

Supplementary Table 27 Associations between traumatic brain injury from birth to age 11 years, with no additional orthopaedic injury, and psychiatric symptoms based on the Development and Well-Being Assessment (DAWBA) at age 15 years compared to orthopaedic injury on complete case sample

DAWBA	Unadjusted OR (95% CI)	Model 1 OR (95% CI)	Model 2 OR (95% CI)
	Externalising Behaviour [a*]		
n	*n = 3063*	*n = 3063*	*n = 3063*
TBI only vs OI	2.25 (0.98 - 5.17)	2.31 (1.01 - 5.32)	1.99 (0.85 - 4.64)
Omnibus p	0.759	0.747	0.698
	ODD [b**]		
n	*n = 3056*	*n = 3056*	*n = 3056*
TBI only vs OI	2.25 (0.93 - 5.44)	2.28 (0.94 - 5.52)	1.94 (0.79 - 4.79)
Omnibus p	0.863	0.880	0.901
	CD [c**]		
n	*n = 3055*	*n = 3055*	*n = 3055*
TBI only vs OI	0.81 (0.10 - 6.54)	0.88 (0.11 - 7.12)	0.67 (0.08 - 5.60)
Omnibus p	0.617	0.624	0.652
	ADHD		
n	*n = 3063*	*n = 3063*	*n = 3063*
TBI only vs OI	4.38 (0.72 - 26.52)	4.77 (0.78 - 29.23)	4.03 (0.65 - 25.14)
Omnibus p	0.585	0.565	0.514

Complete cases had no missing data for the exposure, outcome or covariates. *TBI: traumatic brain injury; OI: orthopaedic injury; * logistic regression; [a] externalising disorder symptoms based on the Development and Well-being Assessment (DAWBA) self-reported at age 15 years; [b] ODD: oppositional defiant disorder based on the Development and Well-being Assessment (DAWBA) self-reported at age 15 years; [c] CD: conduct disorder based on the Development and Well-being Assessment (DAWBA) self-reported at age 15 years; [d] ADHD: attentional defiant hyperactivity disorder based on the Development and Well-being Assessment (DAWBA) self-reported at age 15 years*

Unadjusted: Injuries from birth to age 16 years with main substance use variable in each analysis
Model 1: As unadjusted with additional adjustment for pre-birth confounders (mother's age at birth, mother's education at birth, social class and gender)
Model 2: As Model 1 with additional adjustment for childhood confounders (early life events, parental bonding, positive and negative parenting experiences, maternal alcohol use and maternal tobacco smoking)

Supplementary Table 28 Associations between traumatic brain injury and orthopaedic injuries from age 12 to age 16 years and substance use at 17 years

Substance Use	Unadjusted OR (95% CI)	Model 1 OR (95% CI)	Model 2 OR (95% CI)	Model 3 OR (95% CI)
	Alcohol [a*]			
n	*n = 2926*	*n = 2580*	*n = 2263*	*n = 1649*
TBI vs no Injury	1.71 (1.28 - 2.27)	1.59 (1.17 - 2.15)	1.72 (1.25 - 2.37)	1.41 (0.93 - 2.15)
OI vs no Injury	1.06 (0.83 - 1.35)	0.98 (0.75 - 1.27)	0.98 (0.74 - 1.31)	0.70 (0.47 - 1.04)
TBI vs OI	1.61 (1.13 - 2.31)	1.62 (1.11 - 2.38)	1.76 (1.17 - 2.63)	2.03 (1.17 - 3.53)
Omnibus p	0.116	0.432	0.319	0.282
	Tobacco [b**]			
n	*n = 2488*	*n = 2193*	*n = 1923*	*n = 1649*
TBI vs no Injury	1.56 (1.11 – 2.19)	1.67 (1.16 – 2.41)	1.71 (1.15 - 2.52)	1.15 (0.72 - 1.86)
OI vs no Injury	1.50 (1.13 - 2.00)	1.61 (1.17 - 2.21)	1.76 (1.25 - 2.48)	2.00 (1.29 - 3.09)
TBI vs OI	1.04 (0.68 - 1.58)	1.04 (0.66 - 1.63)	0.97 (0.60 - 1.57)	0.58 (0.32 - 1.06)
Omnibus p	0.001	0.000	0.000	0.003
	Cannabis [c**]			
n	*n = 3172*	*n = 2788*	*n = 2439*	*n = 1649*
TBI vs no Injury	1.49 (1.11 - 1.99)	1.32 (0.97 - 1.81)	1.36 (0.98 - 1.88)	1.14 (0.74 - 1.76)
OI vs no Injury	1.32 (1.03 - 1.68)	1.23 (0.95 - 1.60)	1.28 (0.96 - 1.72)	1.04 (0.69 - 1.58)
TBI vs OI	1.13 (0.79 - 1.62)	1.08 (0.73 - 1.58)	1.06 (0.70 - 1.60)	1.09 (0.62 - 1.92)
Omnibus p	0.004	0.051	0.034	0.702

Sample size reduces per adjustment as the participants who are missing covariate data get excluded. *TBI: traumatic brain injury; OI: orthopaedic injury; *logistic regression; **generalised ordinal regression; [a] alcohol measured using the Alcohol Use Disorder Identification Test (AUDIT); [b] tobacco measured using the Fagerström Test for Nicotine Dependence; [c] cannabis measured using the Cannabis Abuse Screening Test.*

Unadjusted: Injuries from birth to age 16 years with main substance use variable in each analysis
Model 1: As unadjusted with additional adjustment for pre-birth confounders (mother's age at birth, mother's education at birth, social class and gender)
Model 2: As Model 1 with additional adjustment for childhood confounders (early life events, parental bonding, positive and negative parenting experiences, maternal alcohol use and maternal tobacco smoking)
Model 3: As Model 2 with additional adjustment for substance use and crime variables

Supplementary Table 29 Associations between traumatic brain injury from age 12 to age 16 years, with no additional orthopaedic injury, and substance use at age 17 years compared to orthopaedic injury

Substance Use	Unadjusted OR (95% CI)	Model 1 OR (95% CI)	Model 2 OR (95% CI)	Model 3 OR (95% CI)
	Alcohol [a*]			
n	*n = 2890*	*n = 2548*	*n = 2232*	*n = 1625*
TBI only vs OI	1.59 (1.09 - 2.32)	1.58 (1.06 - 2.37)	1.76 (1.14 - 2.71)	2.41 (1.35 - 4.29)
Omnibus p	0.172	0.540	0.403	0.364
	Tobacco [b**]			
n	*n = 2455*	*n = 2164*	*n = 1895*	*n = 1625*
TBI only vs OI	0.95 (0.60 - 1.49)	0.89 (0.54 - 1.47)	0.82 (0.48 - 1.40)	0.59 (0.31 - 1.13)
Omnibus p	0.002	0.001	<0.001	0.002
	Cannabis [c**]			
N	*n = 3135*	*n = 2755*	*n = 2407*	*n = 1625*
TBI only vs OI	1.06 (0.72 - 1.56)	0.997 (0.66 - 1.51)	0.97 (0.62 - 1.51)	1.12 (0.61 - 2.03)
Omnibus p	0.008	0.070	0.051	0.714

Sample size reduces per adjustment as the participants who are missing covariate data get excluded. *TBI: traumatic brain injury; OI: orthopaedic injury; *logistic regression; **generalised ordinal regression; [a] alcohol measured using the Alcohol Use Disorder Identification Test (AUDIT); [b] tobacco measured using the Fagerström Test for Nicotine Dependence; [c] cannabis measured using the Cannabis Abuse Screening Test.*

Unadjusted: Injuries from birth to age 16 years with main substance use variable in each analysis
Model 1: As unadjusted with additional adjustment for pre-birth confounders (mother's age at birth, mother's education at birth, social class and gender)
Model 2: As Model 1 with additional adjustment for childhood confounders (early life events, parental bonding, positive and negative parenting experiences, maternal alcohol use and maternal tobacco smoking)
Model 3: As Model 2 with additional adjustment for substance use and crime variables

Supplementary Table 30 Associations between traumatic brain injury and orthopaedic injuries from age 12 to 16 years and criminal behaviour at age 17 years

Criminal Behaviour	Unadjusted OR (95% CI)	Model 1 OR (95% CI)	Model 2 OR (95% CI)	Model 3 OR (95% CI)
	Offences [a]**			
n	*n = 3079*	*n = 2718*	*n = 2372*	*n = 1681*
TBI vs no Injury	2.05 (1.50 - 2.80)	1.88 (1.34 - 2.63)	1.99 (1.40 - 2.82)	1.52 (0.97 - 2.39)
OI vs no Injury	1.89 (1.44 - 2.45)	1.47 (1.09 - 1.97)	1.53 (1.11 - 2.11)	1.49 (0.99 - 2.24)
TBI vs OI	1.09 (0.74 - 1.60)	1.28 (0.85 - 1.94)	1.30 (0.83 - 2.01)	1.02 (0.58 - 1.79)
Omnibus p	<0.001	0.001	<0.001	0.022
	Trouble with the Police [b]**			
n	*n = 3024*	*n = 2668*	*n = 2339*	*n = 1651*
TBI vs no Injury	1.74 (1.22 – 2.48)	1.43 (0.96 - 2.13)	1.51 (1.00 - 2.29)	1.21 (0.72 - 2.03)
OI vs no Injury	1.59 (1.17 - 2.17)	1.09 (0.77 - 1.54)	1.12 (0.77 - 1.64)	0.86 (0.52 - 1.42)
TBI vs OI	1.09 (0.70 - 1.70)	1.31 (0.80 - 2.15)	1.35 (0.80 - 2.27)	1.41 (0.72 - 2.77)
Omnibus p	<0.001	0.360	0.252	0.756

Sample size reduces per adjustment as the participants who are missing covariate data get excluded. *TBI: traumatic brain injury; OI: orthopaedic injury; **generalised ordinal regression; [a] offences measured by self-report questionnaire at age 17 years; [b] trouble with the police measured by self-report questionnaire at age 17 years.*

Unadjusted: Injuries from birth to age 16 years with main substance use variable in each analysis
Model 1: As unadjusted with additional adjustment for pre-birth confounders (mother's age at birth, mother's education at birth, social class and gender)
Model 2: As Model 1 with additional adjustment for childhood confounders (early life events, parental bonding, positive and negative parenting experiences, maternal alcohol use and maternal tobacco smoking)
Model 3: As Model 2 with additional adjustment for substance use variables

Supplementary Table 31 Associations between traumatic brain injury from age 12 to age 16 years, with no additional orthopaedic injury, and criminal behaviour at age 17 years compared to orthopaedic injury

Criminal Behaviour	Unadjusted<br>OR (95% CI)	Model 1<br>OR (95% CI)	Model 2<br>OR (95% CI)	Model 3<br>OR (95% CI)
	Offences [a]**			
n	*n = 3043*	*n = 2682*	*n = 2341*	*n = 1657*
TBI only vs OI	0.94 (0.62 - 1.42)	1.09 (0.70 - 1.71)	1.09 (0.67 - 1.75)	0.85 (0.46 - 1.58)
Omnibus p	<0.001	0.002	0.002	0.041
	Trouble with the Police [b]**			
n	*n = 2988*	*n = 2636*	*n = 2308*	*n = 1627*
TBI only vs OI	0.80 (0.48 - 1.31)	0.90 (0.51 - 1.58)	0.90 (0.50 - 1.64)	0.92 (0.43 - 1.99)
Omnibus p	0.002	0.662	0.538	0.435

Sample size reduces per adjustment as the participants who are missing covariate data get excluded. *TBI: traumatic brain injury; OI: orthopaedic injury; **generalised ordinal regression; [a] offences measured by self-report questionnaire at age 17 years; [b] trouble with the police measured by self-report questionnaire at age 17 years.*

Unadjusted: Injuries from birth to age 16 years with main substance use variable in each analysis
Model 1: As unadjusted with additional adjustment for pre-birth confounders (mother's age at birth, mother's education at birth, social class and gender)
Model 2: As Model 1 with additional adjustment for childhood confounders (early life events, parental bonding, positive and negative parenting experiences, maternal alcohol use and maternal tobacco smoking)
Model 3: As Model 2 with additional adjustment for substance use variables

Supplementary Table 32 Associations between traumatic brain injury and orthopaedic injuries from age 12 to 16 years and psychiatric symptoms based on the Strengths and Difficulties Questionnaire at age 17 years

SDQ	Unadjusted<br>OR (95% CI)	Model 1<br>OR (95% CI)	Model 2<br>OR (95% CI)
	Conduct problems [a*]		
n	*n = 4460*	*n = 3939*	*n = 3536*
TBI vs no Injury	1.21 (0.74 – 1.96)	1.41 (0.84 – 2.38)	1.39 (0.81 - 2.39)
OI vs no Injury	1.39 (0.92 - 2.12)	1.46 (0.92 - 2.31)	1.37 (0.84 - 2.24)
TBI vs OI	0.87 (0.47 - 1.59)	0.97 (0.50 - 1.85)	1.02 (0.51 - 2.02)
Omnibus p	0.095	0.058	0.126
	Peer Problems [b*]		
n	*n = 4452*	*n = 3929*	*n = 3526*
TBI vs no Injury	0.87 (0.55 – 1.38)	0.75 (0.45 – 1.25)	0.72 (0.42 - 1.24)
OI vs no Injury	0.87 (0.57 - 1.32)	0.56 (0.34 - 0.93)	0.55 (0.32 - 0.95)
TBI vs OI	1.00 (0.55 - 1.81)	1.35 (0.67 - 2.69)	1.30 (0.62 - 2.74)
Omnibus p	0.421	0.014	0.018

Sample size reduces per adjustment as the participants who are missing covariate data get excluded. *TBI: traumatic brain injury; OI: orthopaedic injury; *logistic regression; [a] conduct problems based on parent-completed Strengths and Difficulties Questionnaire at age 17 years; [b] peer problems based on parent-completed Strengths and Difficulties Questionnaire at age 17 years.*

Unadjusted: Injuries from birth to age 16 years with main substance use variable in each analysis
Model 1: As unadjusted with additional adjustment for pre-birth confounders (mother's age at birth, mother's education at birth, social class and gender)
Model 2: As Model 1 with additional adjustment for childhood confounders (early life events, parental bonding, positive and negative parenting experiences, maternal alcohol use and maternal tobacco smoking)

Supplementary Table 33 Associations between traumatic brain injury from age 12 to age 16 years, with no additional orthopaedic injury, and psychiatric symptoms at age 17 years compared to orthopaedic injury

SDQ	Unadjusted<br>OR (95% CI)	Model 1<br>OR (95% CI)	Model 2<br>OR (95% CI)
	Conduct problems [a*]		
n	*n = 4404*	*n = 3890*	*n = 3490*
TBI only vs OI	1.01 (0.54 - 1.87)	1.13 (0.58 - 2.19)	1.18 (0.58 - 2.38)
Omnibus p	0.065	0.042	0.096
	Peer Problems [b*]		
n	*n = 4396*	*n = 3880*	*n = 3480*
TBI only vs OI	1.17 (0.64 - 2.14)	1.58 (0.78 - 3.21)	1.51 (0.71 - 3.22)
Omnibus p	0.560	0.024	0.029

Sample size reduces per adjustment as the participants who are missing covariate data get excluded. *TBI: traumatic brain injury; OI: orthopaedic injury; *logistic regression; [a] conduct problems based on parent-completed Strengths and Difficulties Questionnaire at age 17 years; [b] peer problems based on parent-completed Strengths and Difficulties Questionnaire at age 17 years.*

Unadjusted: Injuries from birth to age 16 years with main substance use variable in each analysis
Model 1: As unadjusted with additional adjustment for pre-birth confounders (mother's age at birth, mother's education at birth, social class and gender)
Model 2: As Model 1 with additional adjustment for childhood confounders (early life events, parental bonding, positive and negative parenting experiences, maternal alcohol use and maternal tobacco smoking)

Supplementary Table 34 Associations between traumatic brain injury and orthopaedic injuries from age 12 to age 16 years and substance use at 17 years on complete case sample

Substance Use	Unadjusted<br>OR (95% CI)	Model 1<br>OR (95% CI)	Model 2<br>OR (95% CI)	Model 3<br>OR (95% CI)
	Alcohol [a*]			
n	*n = 1649*	*n = 1649*	*n = 1649*	*n = 1649*
TBI vs no Injury	1.76 (1.22 - 2.54)	1.72 (1.19 - 2.49)	1.71 (0.60 - 1.20)	1.41 (0.93 - 2.15)
OI vs no Injury	0.88 (0.63 - 1.24)	0.84 (0.59 - 1.18)	0.85 (0.60 - 1.20)	0.70 (0.47 - 1.04)
TBI vs OI	2.00 (1.24 - 3.21)	2.05 (1.27 - 3.31)	2.02 (1.24 - 3.27)	2.03 (1.17 - 3.53)
Omnibus p	0.721	0.964	0.915	0.282
	Tobacco [b**]			
n	*n = 1649*	*n = 1649*	*n = 1649*	*n = 1649*
TBI vs no Injury	1.55 (1.04 - 2.31)	1.70 (1.13 - 2.55)	1.69 (1.12 - 2.55)	1.15 (0.72 - 1.86)
OI vs no Injury	1.55 (1.08 - 2.22)	1.60 (1.10 - 2.31)	1.68 (1.15 - 2.44)	2.00 (1.29 - 3.09)
TBI vs OI	1.00 (0.60 - 1.65)	1.06 (0.64 - 1.77)	1.01 (0.60 - 1.69)	0.58 (0.32 - 1.06)
Omnibus p	0.004	0.002	0.001	0.003
	Cannabis [c**]			
n	*n = 1649*	*n = 1649*	*n = 1649*	*n = 1649*
TBI vs no Injury	1.71 (1.17 - 2.48)	1.64 (1.12 - 2.39)	1.64 (1.12 - 2.41)	1.14 (0.74 - 1.76)
OI vs no Injury	1.26 (0.89 - 1.78)	1.18 (0.83 - 1.68)	1.18 (0.83 - 1.69)	1.04 (0.69 - 1.58)
TBI vs OI	1.35 (0.84 - 2.19)	1.39 (0.85 - 2.26)	1.39 (0.85 - 2.27)	1.09 (0.62 - 1.92)
Omnibus p	0.035	0.094	0.092	0.702

Complete cases had no missing data for the exposure, outcome or covariates*. TBI: traumatic brain injury; OI: orthopaedic injury; * logistic regression; **generalised ordinal regression; [a] alcohol measured using the Alcohol Use Disorder Identification Test (AUDIT); [b] tobacco measured using the Fagerström Test for Nicotine Dependence; [c] cannabis measured using the Cannabis Abuse Screening Test.*

Unadjusted: Injuries from birth to age 16 years with main substance use variable in each analysis
Model 1: As unadjusted with additional adjustment for pre-birth confounders (mother's age at birth, mother's education at birth, social class and gender)
Model 2: As Model 1 with additional adjustment for childhood confounders (early life events, parental bonding, positive and negative parenting experiences, maternal alcohol use and maternal tobacco smoking)
Model 3: As Model 2 with additional adjustment for substance use and crime variables

Supplementary Table 35 Associations between traumatic brain injury from age 12 to age 16 years, with no additional orthopaedic injury, and substance use at age 17 years compared to orthopaedic injury on complete case sample

Substance Use	Unadjusted OR (95% CI)	Model 1 OR (95% CI)	Model 2 OR (95% CI)	Model 3 OR (95% CI)
	Alcohol [a*]			
n	*n = 1625*	*n = 1625*	*n = 1625*	*n = 1625*
TBI only vs OI	2.07 (1.25 - 3.43)	2.13 (1.28 - 3.55)	2.09 (1.25 - 3.49)	2.41 (1.35 - 4.29)
Omnibus p	0.823	0.952	0.997	0.364
	Tobacco [b**]			
n	*n = 1625*	*n = 1625*	*n = 1625*	*n = 1625*
TBI only vs OI	0.83 (0.47 - 1.44)	0.89 (0.51 - 1.56)	0.84 (0.48 - 1.49)	0.59 (0.31 - 1.13)
Omnibus p	0.011	0.006	0.003	0.002
	Cannabis [c**]			
n	*n = 1625*	*n = 1625*	*n = 1625*	*n = 1625*
TBI only vs OI	1.16 (0.69 - 1.95)	1.21 (0.72 - 2.05)	1.21 (0.71 - 2.06)	1.12 (0.61 - 2.03)
Omnibus p	0.081	0.164	0.163	0.714

Complete cases had no missing data for the exposure, outcome or covariates*. TBI: traumatic brain injury; OI: orthopaedic injury; * logistic regression; **generalised ordinal regression; [a] alcohol measured using the Alcohol Use Disorder Identification Test (AUDIT); [b] tobacco measured using the Fagerström Test for Nicotine Dependence; [c] cannabis measured using the Cannabis Abuse Screening Test.*

Unadjusted: Injuries from birth to age 16 years with main substance use variable in each analysis
Model 1: As unadjusted with additional adjustment for pre-birth confounders (mother's age at birth, mother's education at birth, social class and gender)
Model 2: As Model 1 with additional adjustment for childhood confounders (early life events, parental bonding, positive and negative parenting experiences, maternal alcohol use and maternal tobacco smoking)
Model 3: As Model 2 with additional adjustment for substance use and crime variables

Supplementary Table 36 Associations between traumatic brain injury and orthopaedic injuries from age 12 to 16 years and criminal behaviour at age 17 years on complete case sample

Criminal Behaviour	Unadjusted OR (95% CI)	Model 1 OR (95% CI)	Model 2 OR (95% CI)	Model 3 OR (95% CI)
	Offences [a]**			
n	*n = 1681*	*n = 1681*	*n = 1681*	*n = 1681*
TBI vs no Injury	2.22 (1.49 - 3.30)	2.02 (1.35 - 3.02)	2.01 (1.34 - 3.01)	1.52 (0.97 - 2.39)
OI vs no Injury	2.01 (1.40 - 2.88)	1.59 (1.10 - 2.30)	1.59 (1.10 - 2.30)	1.49 (0.99 - 2.24)
TBI vs OI	1.10 (0.67 - 1.81)	1.27 (0.76 - 2.10)	1.27 (0.76 - 2.11)	1.02 (0.58 - 1.79)
Omnibus p	<0.001	0.001	0.001	0.022
	Trouble with the Police [b]**			
n	*n = 1651*	*n = 1651*	*n = 1651*	*n = 1651*
TBI vs no Injury	1.89 (1.22 – 2.94)	1.75 (1.11 - 2.76)	1.74 (1.10 - 2.76)	1.21 (0.72 - 2.03)
OI vs no Injury	1.26 (0.81 - 1.95)	0.90 (0.57 - 1.42)	0.91 (0.57 - 1.43)	0.86 (0.52 - 1.42)
TBI vs OI	1.51 (0.84 - 2.70)	1.94 (1.06 - 3.55)	1.92 (1.05 - 3.53)	1.41 (0.72 - 2.77)
Omnibus p	0.063	0.751	0.728	0.756

Complete cases had no missing data for the exposure, outcome or covariates*. TBI: traumatic brain injury; OI: orthopaedic injury; **generalised ordinal regression; [a] offences measured by self-report questionnaire at age 17 years; [b] trouble with the police measured by self-report questionnaire at age 17 years.*

Unadjusted: Injuries from birth to age 16 years with main substance use variable in each analysis
Model 1: As unadjusted with additional adjustment for pre-birth confounders (mother's age at birth, mother's education at birth, social class and gender)
Model 2: As Model 1 with additional adjustment for childhood confounders (early life events, parental bonding, positive and negative parenting experiences, maternal alcohol use and maternal tobacco smoking)
Model 3: As Model 2 with additional adjustment for substance use variables

Supplementary Table 37 Associations between traumatic brain injury from age 12 to age 16 years, with no additional orthopaedic injury, and criminal behaviour at age 17 years compared to orthopaedic injury on complete case sample

Criminal Behaviour	Unadjusted OR (95% CI)	Model 1 OR (95% CI)	Model 2 OR (95% CI)	Model 3 OR (95% CI)
	Offences [a]**			
n	*n = 1657*	*n = 1657*	*n = 1657*	*n = 1657*
TBI only vs OI	0.87 (0.50 - 1.50)	1.03 (0.59 - 1.79)	1.03 (0.59 - 1.80)	0.85 (0.46 - 1.58)
Omnibus p	<0.001	0.004	0.004	0.041
	Trouble with the Police [b]**			
n	*n = 1627*	*n = 1627*	*n = 1627*	*n = 1627*
TBI only vs OI	0.91 (0.47 - 1.79)	1.20 (0.60 - 2.41)	1.19 (0.59 - 2.38)	0.92 (0.43 - 1.99)
Omnibus p	0.277	0.744	0.767	0.435

Complete cases had no missing data for the exposure, outcome or covariates*. TBI: traumatic brain injury; OI: orthopaedic injury; **generalised ordinal regression; [a] offences measured by self-report questionnaire at age 17 years; [b] trouble with the police measured by self-report questionnaire at age 17 years.*

Unadjusted: Injuries from birth to age 16 years with main substance use variable in each analysis
Model 1: As unadjusted with additional adjustment for pre-birth confounders (mother's age at birth, mother's education at birth, social class and gender)
Model 2: As Model 1 with additional adjustment for childhood confounders (early life events, parental bonding, positive and negative parenting experiences, maternal alcohol use and maternal tobacco smoking)
Model 3: As Model 2 with additional adjustment for substance use variables

Supplementary Table 38 Associations between traumatic brain injury and orthopaedic injuries from age 12 to 16 years and psychiatric symptoms based on the Strengths and Difficulties Questionnaire at age 17 years on complete case sample

SDQ	Unadjusted<br>OR (95% CI)	Model 1<br>OR (95% CI)	Model 2<br>OR (95% CI)
	Conduct problems [a*]		
n	*n = 3536*	*n = 3536*	*n = 3536*
TBI vs no Injury	1.31 (0.77 – 2.24)	1.41 (0.83 – 2.41)	1.39 (0.81 - 2.39)
OI vs no Injury	1.35 (0.83 - 2.19)	1.37 (0.84 – 2.24)	1.37 (0.84 - 2.24)
TBI vs OI	0.97 (0.49 - 1.91)	1.03 (0.52 - 2.03)	1.02 (0.51 - 2.02)
Omnibus p	0.147	0.116	0.126
	Peer Problems [b*]		
n	*n = 3526*	*n = 3526*	*n = 3526*
TBI vs no Injury	0.76 (0.44 – 1.30)	0.71 (0.41 – 1.22)	0.72 (0.42 - 1.24)
OI vs no Injury	0.62 (0.36 - 1.06)	0.55 (0.32 - 0.94)	0.55 (0.32 - 0.95)
TBI vs OI	1.22 (0.59 - 2.55)	1.29 (0.62 - 2.70)	1.30 (0.62 - 2.74)
Omnibus p	0.053	0.015	0.018

Complete cases had no missing data for the exposure, outcome or covariates. *TBI: traumatic brain injury; OI: orthopaedic injury; *logistic regression; [a] conduct problems based on parent-completed Strengths and Difficulties Questionnaire at age 17 years; [b] peer problems based on parent-completed Strengths and Difficulties Questionnaire at age 17 years.*

Unadjusted: Injuries from birth to age 16 years with main substance use variable in each analysis
Model 1: As unadjusted with additional adjustment for pre-birth confounders (mother's age at birth, mother's education at birth, social class and gender)
Model 2: As Model 1 with additional adjustment for childhood confounders (early life events, parental bonding, positive and negative parenting experiences, maternal alcohol use and maternal tobacco smoking)

Supplementary Table 39 Associations between traumatic brain injury from age 12 to age 16 years, with no additional orthopaedic injury, and psychiatric symptoms at age 17 years compared to orthopaedic injury on complete case sample

SDQ	Unadjusted<br>OR (95% CI)	Model 1<br>OR (95% CI)	Model 2<br>OR (95% CI)
	Conduct problems [a]*		
n	*n = 3490*	*n = 3490*	*n = 3490*
TBI only vs OI	1.13 (0.56 - 2.25)	1.2 (0.60 - 2.42)	1.18 (0.58 - 2.38)
Omnibus p	0.111	0.088	0.096
	Peer Problems [b]*		
n	*n = 3480*	*n = 3480*	*n = 3480*
TBI only vs OI	1.41 (0.66 - 2.98)	1.51 (0.71 - 3.21)	1.51 (0.71 - 3.22)
Omnibus p	0.079	0.025	0.029

Complete cases had no missing data for the exposure, outcome or covariates. *TBI: traumatic brain injury; OI: orthopaedic injury; *logistic regression; [a] conduct problems based on parent-completed Strengths and Difficulties Questionnaire at age 17 years; [b] peer problems based on parent-completed Strengths and Difficulties Questionnaire at age 17 years.*

Unadjusted: Injuries from birth to age 16 years with main substance use variable in each analysis
Model 1: As unadjusted with additional adjustment for pre-birth confounders (mother's age at birth, mother's education at birth, social class and gender)
Model 2: As Model 1 with additional adjustment for childhood confounders (early life events, parental bonding, positive and negative parenting experiences, maternal alcohol use and maternal tobacco smoking)
